# Intramuscular mRNA BNT162b2 vaccine against SARS-CoV-2 induces neutralizing salivary IgA

**DOI:** 10.3389/fimmu.2022.933347

**Published:** 2023-01-30

**Authors:** Miri Stolovich-Rain, Sujata Kumari, Ahuva Friedman, Saveliy Kirillov, Yakov Socol, Maria Billan, Ritesh Ranjan Pal, Kathakali Das, Peretz Golding, Esther Oiknine-Djian, Salim Sirhan, Michal Bejerano Sagie, Einav Cohen-Kfir, Naama Gold, Jamal Fahoum, Manoj Kumar, Maya Elgrably-Weiss, Bing Zhou, Miriam Ravins, Yair E. Gatt, Saurabh Bhattacharya, Orly Zelig, Reuven Wiener, Dana G. Wolf, Hila Elinav, Jacob Strahilevitz, Dan Padawer, Leah Baraz, Alexander Rouvinski

**Affiliations:** ^1^ Department of Microbiology and Molecular Genetics, Institute for Medical Research Israel-Canada, The Hebrew University-Hadassah Medical School, The Hebrew University of Jerusalem, Jerusalem, Israel; ^2^ The Kuvin Center for the Study of Infectious and Tropical Diseases, The Hebrew University-Hadassah Medical School, The Hebrew University of Jerusalem, Jerusalem, Israel; ^3^ Department of Developmental Biology and Cancer Research, Institute for Medical Research Israel-Canada, The Hebrew University-Hadassah Medical School, The Hebrew University of Jerusalem, Jerusalem, Israel; ^4^ Department of Biochemistry, Institute for Medical Research Israel-Canada, The Hebrew University-Hadassah Medical School, The Hebrew University of Jerusalem, Jerusalem, Israel; ^5^ National Center for Biotechnology, Astana, Kazakhstan; ^6^ Department of General Biology and Genomics, L.N. Gumilyov Eurasian National University, Astana, Kazakhstan; ^7^ Clinical Virology Unit, Hadassah Hebrew University Medical Center, Jerusalem, Israel Hadassah Hebrew University Medical Center, Jerusalem, Israel; ^8^ Blood Bank, Hadassah Hebrew University Medical Center, Jerusalem, Israel; ^9^ Lautenberg Centre for Immunology and Cancer Research, The Institute for Medical Research Israel-Canada (IMRIC), Faculty of Medicine, Hebrew University of Jerusalem, Jerusalem, Israel; ^10^ Department of Clinical Microbiology and Infectious Diseases, Hadassah AIDS Center, Hadassah Hebrew University Medical Center, Jerusalem, Israel; ^11^ Department of Clinical Microbiology and Infectious Diseases, Hadassah Hebrew University Medical Center, Jerusalem, Israel; ^12^ Institute of Pulmonary Medicine, Hadassah Medical Center, Affiliated to the Faculty of Medicine, Hebrew University Jerusalem, Jerusalem, Israel; ^13^ Department of Internal Medicine D, Hadassah Medical Center, affiliated to the Faculty of Medicine, Hebrew University, Jerusalem, Israel; ^14^ Hadassah Academic College Jerusalem, Jerusalem, Israel

**Keywords:** secretory IgA, mucosal immunity, secretory component, BNT162b2 vaccine, SARS-CoV-2 neutralizing Abs

## Abstract

Intramuscularly administered vaccines stimulate robust serum neutralizing antibodies, yet they are often less competent in eliciting sustainable “sterilizing immunity” at the mucosal level. Our study uncovers a strong temporary neutralizing mucosal component of immunity, emanating from intramuscular administration of an mRNA vaccine. We show that saliva of BNT162b2 vaccinees contains temporary IgA targeting the receptor-binding domain (RBD) of severe acute respiratory syndrome coronavirus-2 spike protein and demonstrate that these IgAs mediate neutralization. RBD-targeting IgAs were found to associate with the secretory component, indicating their *bona fide* transcytotic origin and their polymeric multivalent nature. The mechanistic understanding of the high neutralizing activity provided by mucosal IgA, acting at the first line of defense, will advance vaccination design and surveillance principles and may point to novel treatment approaches and new routes of vaccine administration and boosting.

## Highlights

We revealed strong mucosal neutralization upon BNT162b2 vaccination, mediated by temporary polymeric IgA, and explored its longitudinal properties. We describe immunological characteristics and kinetics of the IgG and IgA response and of its mucosal component upon mRNA vaccination with BNT162b2. We suggest a methodology for quantitative comparison of immunoreactivity and neutralization for IgG and IgA in serum and saliva in molar equivalents that may apply for standardization in diagnostics, for surveillance of protection, and for vaccine evaluations.

## Introduction

Sterilizing immunity is defined as the ability of the immune system to prevent massive replication and subsequent transmission of a pathogen. Primary infection of some viral pathogens at mucosal surfaces is capable of eliciting sterilizing mucosal and systemic immunity, which is activated in a case of secondary exposure (e.g., enteric polio- and rotaviruses, as well as respiratory Influenza virus). Mimicry of certain elements of viral infection by vaccination aims to train the immune system to be tuned for subsequent challenges with the actual pathogen (1). An ultimate goal of a vaccination campaign, in addition to protection against the disease and death, is to achieve a robust sterilizing effect, alleviate the carrier state, and interrupt the transmission cycle in the population (2). In this view, vaccine efficiency has several distinct, albeit interconnected, aspects: (i) reduction of viral load at the entry site and prevention ot the spread between individuals, (ii) prevention of viral spread within the host and expedition viral clearance, and (iii) protection from symptoms or reduction of disease severity.

During the natural course of viral infections pre-symptomatic and asymptomatic individuals can transmit the virus. As an analogy, a vaccine that protects from the disease does not necessarily achieve the sterilizing effect. The presence of pathogen-targeting IgA at mucosal surfaces is known to correlate with sterilizing immunity, thereby preventing transmission of respiratory and enteric viruses (3–9).

Severe acute respiratory syndrome coronavirus-2 (SARS-CoV-2), the etiological agent of coronavirus disease 2019 (COVID-19), is a highly contagious and difficult to contain respiratory virus, regardless of disease status and severity. This is in part because in addition to symptomatic transmission, asymptomatic and pre-symptomatic individuals are able to transmit the infection (10, 11). The two mRNA COVID-19 vaccines, BNT162b2 (Pfizer/BioNtech) and mRNA-1273 (Moderna), have successfully reduced the burden of symptomatic COVID-19 and its more serious outcomes, e.g (12–14). However, the emerging SARS-CoV-2 variants raise concerns as to its long-term protective capability. The immunoglobulin gamma (IgG) responses to natural SARS-CoV-2 infection and the role of IgG against the spike protein and its receptor-binding domain (RBD) in virus neutralization and disease prevention are well established, e.g (9, 15–17). The IgG response to the vaccine has also been reported thoroughly reported, e.g (18–20).

IgA is the most abundant immunoglobulin isotype in humans with daily secreted amounts reaching 60 mg/kg/day (21–24). IgA plays a key role in the interaction between the immune system and environmental insults to provide mucosal protection, often serving as the first line of defense (23, 25–28). Beyond its documented role at mucosal surfaces, IgA is the second most abundant isotype in the blood circulation following IgG, comprising about 20% of total circulatory immunoglobulin content. Serum IgA at a steady state is predominantly a monomer, whereas secreted IgA at mucosal surfaces appears in a dimer/polymer form (25, 29–31). The IgA dimer/polymer, joined through the J chain *via* disulfide bridges, forms a secretory component (SC-IgA) together with a portion of the polymeric immunoglobulin receptor (pIgR), which is necessary for trans-epithelial secretion (6, 32–34). Still, in some cases monomeric IgA can be found at mucosa, and multimeric IgA has been reported in serum as well (22). Although IgA-producing B cells in circulation and secondary lymphoid organs are capable of producing polymeric IgA joined by J-chain, its presence in serum is shortlived after a stimulation period. Short half-life of polymeric IgA in serum compared with monomeric IgA is most likely due to clearance by liver hepatocytes (22, 24, 31, 35). Importantly, the mechanistic relationship between mucosal and systemic immunoglobulin responses is not fully resolved (36). Both monomeric and dimeric RBD-targeting IgA elicited by SARS-CoV-2 infection were shown to possess strong neutralizing potential in biological fluids and when tested in a monoclonal Ab setup (37–41). However, the immunological characteristics and kinetics of the IgA response upon mRNA vaccination have not yet been deeply investigated, particularly with regard to its mucosal component (20, 42–48).

Here, we analyzed the humoral immune response to the BNT162b2 vaccine and detected transitory secretory polymeric IgA, which targets the RBD of SARS-CoV-2 spike in the saliva of vaccinees. We unveiled the neutralizing activity of this humoral component of the mucosal defense and explored its kinetic profile. Furthermore, we established a methodology for quantitative comparison of immunoreactivity and neutralization for the humoral IgG and IgA response in serum and saliva in molar equivalents. We submit an approach for the assessment of antibody response that can be applied globally and will ease standardization in diagnostics and surveillance practices, in decision-making in patients’ care, and in comparative vaccine evaluations.

## Results

### Kinetic profiling of circulatory IgG and IgA immunoglobulins in BNT162b2 vaccinees

In the course of monitoring the kinetics of the serological response in a BNT162b2-vaccinated cohort we noticed that, along with a well-characterized IgG response toward RBD, a substantial proportion of vaccinees developed a time-dependent accrual of RBD-targeting IgA. To further understand functional aspects of BNT162b2 protection we studied serum immunoglobulin responses to the vaccine and their kinetic properties. Serum samples were taken from 90 participants (**Table S1**, cohort details), including a pre-COVID-19 cohort, COVID-19 convalescents, and vaccinees aged 24 to 75 who received two BNT162b2 doses at a 3-week interval (time points included pre-vaccination and follow up until 6-month after the first vaccine dose). A more detailed longitudinal follow-up cohort included serum samples (N = 76) collected from 18 participants (**Table S2**, cohort details).

We focused on IgG and IgA directed against the RBD region of the viral spike, as many studies have shown that anti-RBD Abs hamper SARS-CoV-2 entry into host cells by competing with the binding to the host-cell receptor angiotensin-converting enzyme 2 (49–53). To measure the antibody response we produced fully glycosylated recombinant SARS-CoV-2 RBD in a mammalian expression system and used a custom enzyme-linked immunosorbent assay (ELISA) amenable to quantitative measurements (see **Figures S1A–C**, Material and Methods, and **Supplementary Materials** for details and validation).

**Figure 1:**
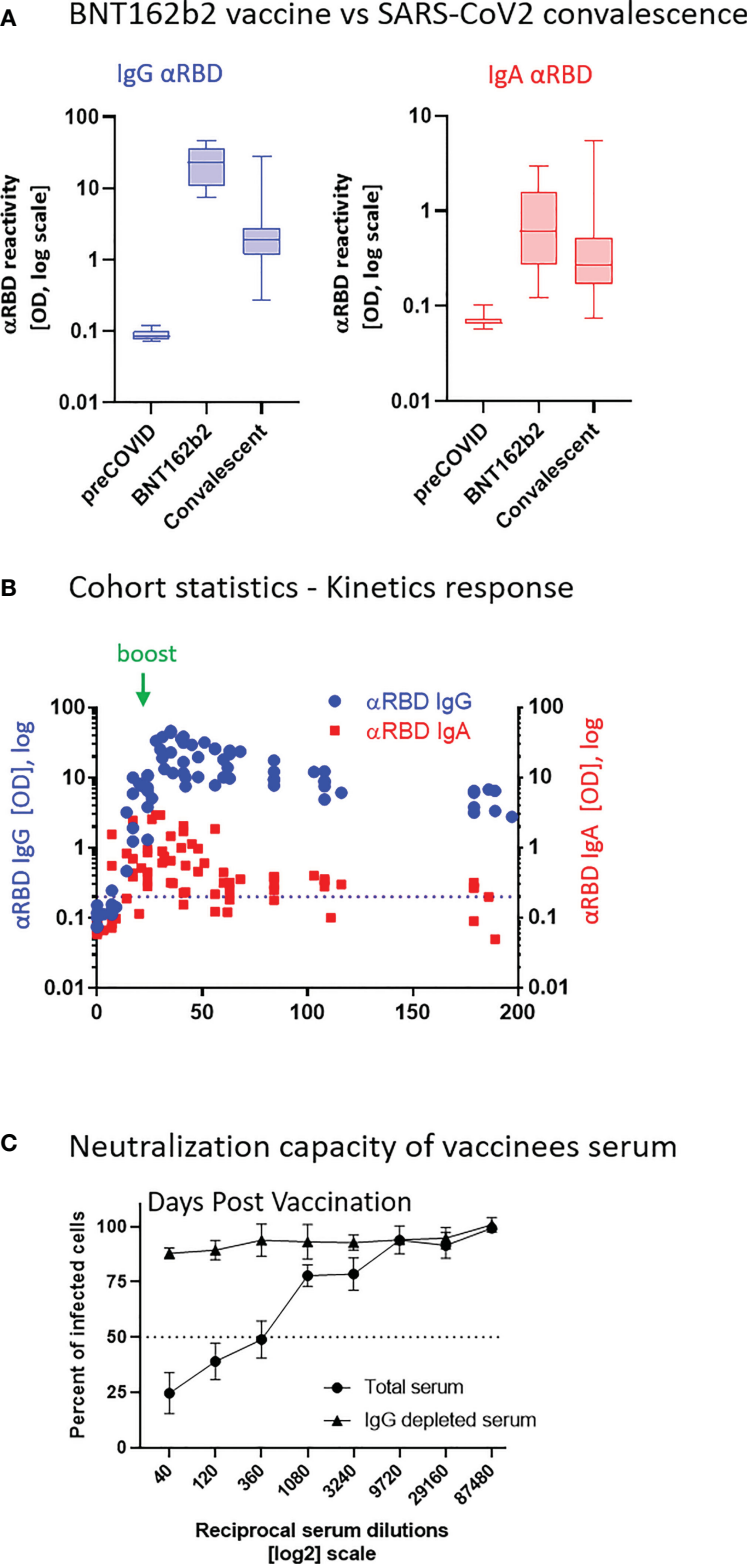
BNT162b2 vaccinees mount serum anti–RBD–SARS-CoV-2 IgG and IgA, with IgG showing strong neutralization potential. **(A)** Independent ELISA measurements of anti-RBD IgG and of anti-RBD IgA in serum samples collected from pre-COVID (N = 51), BNT162b2 vaccinees (N = 17), and post-COVID-19 (N = 22) convalescents, as indicated. Convalescent samples were collected within 3–10 weeks post-recovery, as defined by the clinical definition in Israel at time of sample collection. BNT162b2 vaccinee samples for which kinetic samples were available, the value shown represents the peaks of individual responses based on their kinetic curve [see **(B)** and **Table S7**]. **(B)** Quantitative kinetic profile of anti-RBD IgG (blue) and IgA (red) in serum sampled (N = 76) in the vaccinee cohort (N = 18), plotted as a function of days, after first vaccine dose. See **Table S1** for cohort and sampling details. Independent ordinate axes for IgG (left, blue) and IgA (right, red) highlight the restricted, relative nature of the comparison between isotypes in this experiment, as discussed in the text; see also **Figure 2** for subsequent developments. Green arrows indicate timing of the second vaccine dose (the boost). **(C)** Serum neutralization assessed by SARS-CoV-2 spike-pseudotyped VSV-GFP-ΔG reporter assay on Vero-E6 cells. Neutralization is expressed as a percentage of pseudovirus-infected green cells without serum (total infection = 100%). Percentage of neutralization by sera of pool of four individual vaccinees is plotted as a function of the reciprocal values of sera dilutions displayed on a log2 scale, as indicated (filled circles, total serum, NT50 is reached on average at the dilution of ∼1:360, extrapolated by cross-section with the dashed line. Contribution of IgG to serum neutralization is evaluated by the depletion of the IgG isotype using anti-IgG specific magnetic beads (triangles). See **Figure S1F** for assessment of completeness and specificity of IgG depletion. Results of three experimental repeats are represented.

Robust anti-RBD IgG and IgA activity was evident in all vaccinees at 10–30 days after the second vaccine dose vs. naïve (pre-COVID-19) individuals (**Figure 1A**). Overall, vaccine-induced anti-RBD IgG was stronger than in convalescents, whereas the IgA levels were comparable between the two groups, in agreement with recent reports (54–56). This suggests that, at least from a quantitative perspective, BNT162b2 vaccine prime/boost regimen initiates an anti-RBD humoral immune response in circulation comparable to or even stronger than the one observed upon recovery from natural infection.

Next, we carried out a detailed time course analysis of the serological response among the vaccinees. Notably, the fact that the majority of commercially available SARS-CoV-2 antibody assays, as well as our results presented in **Figure 1B**, use arbitrary unit values impedes the capability to directly compare anti-RBD IgG and IgA levels in terms of molecular stoichiometry. Therefore, we used two different ordinate axes to represent IgG and IgA arbitrary values and only relatively superpose the respective shape and durability of the two isotypes. The magnitude of the serological IgA response among vaccinees was significantly more variable and overall showed a less steep increase than that of the IgG (**Figure 1B**), suggesting a higher variability of the vaccine in inducing the IgA isotype in circulation. Monitoring of the circulatory levels for 6 months after vaccination in a cohort subset revealed a decline of anti-RBD IgG and IgA (**Figure 1B**; see also **Figure S1E** for violin plot representation of categorized periods).

**Figure 2:**
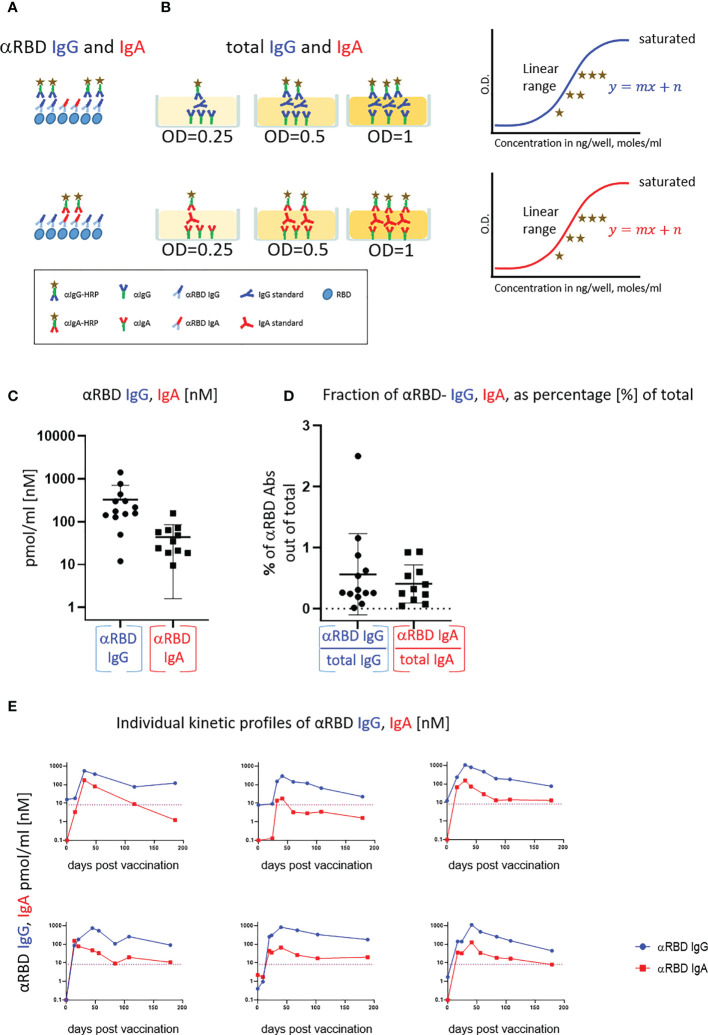
Quantitative ELISA measurement of anti-RBD IgG and IgA content in biological fluids. **(A)** Schematic representation of detection of anti-RBD IgG or IgA by indirect ELISA using isotype-specific HRP-conjugated secondary antibodies. OD values are not directly comparable between the isotypes because of the use of different secondary antibodies. **(B)** Schematic representation of sandwich capturing ELISA for selective quantification of total immunoglobulin isotypes (IgG vs. IgA). Yellow stars schematically represent molar equivalents of antibody quantities. We introduce pure IgA and IgG commercial references to transform the OD values to their molar equivalents, using the standard dilution curve in capture ELISA format. Implementing such a standard in every experiment allows for determining the antigen specific and total molar amounts of each isotype within the linearity range. We assume average molecular weight (MW) of IgG = 146 kDa and IgA = 150 kDa in circulation. **(C)** Molar measurement of anti-RBD IgG and IgA implementing the methodology described in **(A, B)** depicts stoichiometric ratios between the antigen-specific isotypes. **(D)** Percentage of antigen-specific anti-RBD out of total immunoglobulin isotype, as indicated. **(E)** Individual longitidual profiles of anti-RBD IgG (blue) and IgA (red) monitored in six vaccinated individuals up to 7 months after vaccination are inferred as picomole per ml of serum.

To assess the specific functional contributions of IgA and IgG in serum, we performed neutralization analyses using a reporter assay in Vero E6 cells, based on SARS-CoV-2 spike-pseudotyped vesicular stomatitis virus (VSV). First, we selected a pool of sera from four vaccinated individuals with significant anti-RBD IgA and IgG levels and depleted total IgG molecules. **Figure S1F** shows the complete drop in total IgG levels upon depletion, as measured by sandwich ELISA (depicted in **Figure 2B**). Whereas the original sera pool showed the half neutralization capacity (NT50) at the dilution of ∼1:360, the IgG-depleted pool resulted in a complete loss of neutralization (**Figure 1C**). This indicates that the vaccine-elicited IgG is the functionally predominant neutralizing isotype in blood circulation, in accordance with previous findings (55, 57, 58).

### Measuring absolute and proportional amounts of vaccine-elicited IgA and IgG

BNT162b2 vaccine–elicited circulatory anti-RBD IgA drew our attention, particularly due to the crucial importance of IgA in providing the ultimately desired mucosal defense.

As experience with intramuscular RNA vaccination is limited, especially with respect to IgA response overall and in particular at the mucosal interface, we decided to explore the role of secretory IgA. One notable obstacle in the functional assessment of the role of IgA in both circulation and mucosal surfaces is the inability to quantify and compare circulatory and mucosal IgG vs. IgA. In our view, this is of utmost importance for SARS-CoV-2 studies, in particular due to the current need for a universal absolute measure of humoral response at different physiological sites (59). We tackled this obstacle at three levels:

1. Serial dilutions of the serologically evaluated biological fluids to empirically determine the linear confidence range in immunoassays. Samples that fall outside of the linear confidence range are diluted until they can be properly detected.
2. Implementation of pure human IgG and IgA fractions to create a calibration curve to convert optical density (OD) of secondary detection into absolute quantitative units (e.g., moles) independent of the secondary Ab conjugates. This is important for our study, as we compare different biological fluids, IgG and IgA isotypes, and even secretory IgA (see below). This would not be possible using the “spike-in” method described by others when using the commercial antigen tests, many of which are not easily adaptable to the varying titers of antibody present in different fluids. “Spike-in” also relies on a given monoclonal antibody or on a purified polyclonal mixture with a given affinity to the antigen, whereas our method is less biased in this respect. In addition, recombinant SC containing dimeric/polymeric IgA monoclonal antibodies is not even available to the best of our knowledge. Thus, although the classical “spike-in” approach has multiple benefits with regard to the usage of defined recombinant standards, it would not be easily applicable to our study (56, 60).
3. Evaluation of the specific contribution of anti-RBD IgA or IgG by determining their proportion out of total immunoglobulins of the same isotype in a given biological fluid and a subsequent functional assessment of the isotype-specific depletion. We used serum samples of vaccinated individuals described in **Figure 1B** to establish and validate such measurements (see **Table S1** for the sub-cohort details).

In a typical ELISA used to measure RBD antibodies, we coat the plate with RBD and subsequently react it with the relevant biological fluids. RBD-reacting Abs of all isotypes are captured, whereas IgAs and IgGs are then differentially revealed by the corresponding isotype-specific secondary Abs (**Figure 2A**). Using commercial pure human IgG and IgA standards with defined concentrations, we established an “OD-to-mole” transformation (**Figure 2B**). To this end, we use an ELISA setup, measuring the total IgG and IgA populations rather than antigen-specific subsets. In this case, the plate is first coated by capturing isotype-specific Abs and then the defined amounts of the reference isotypes are entrapped and revealed by the isotype-specific Ab-HRP (horseradish peroxidase) secondary conjugates. By introducing such a standard in our experimental routine, we could quantitatively relate OD to the absolute amount of captured immunoglobulins (**Figures S2A–C**, **S5**). **Figure S2D** shows the specificity of isotype capturing and detection, with no apparent cross-reaction.

We next applied this approach to evaluate molar concentrations of RBD-targeting IgG and IgA in the serum of vaccinees. **Figure 2C** quantitatively shows that the majority of individuals produced 200–1,000 pmol/ml (nM) of RBD-targeting IgG vs. 30–200 pmol/ml (nM) of RBD-targeting IgA. Our approach allowed the determination of the proportion of RBD-specific Abs out of the total amount of immunoglobulins of the given isotype in serum (normalized, proportional formula [IgARBD][IgATotal] ) (**Figure 2D**). Whereas absolute quantities of RBD-specific IgG strongly dominate over the corresponding IgA in serum, the normalized fractional quantities were comparable and comprise 0.5% of IgG and 0.4% of IgA in circulation (**Figure 2D**, graphical representation and **Table S4**, sub-cohort data). This near equal proportional representation of both isotypes toward the RBD antigen upon intramuscular mRNA vaccination suggests a similar frequency of class-switch events. In the next series of experiments, we monitored IgA and IgG kinetics expressed in pmol/ml (nM) values of anti-RBD in the serum of six individual vaccinees, allowing stoichiometric comparison (**Figure 2E**). All the individuals exhibited a predominant IgG response that peaked approximately 40 days after vaccination and gradually declined during six months. In contrast, IgA responses were more variable (**Figure 2E**). In all our measurements, the circulatory IgA picomole per milliliter values were lower and with shorter duration than those of IgG, similar to other vaccine instances and upon natural immune responses to infections (61). Of note, for our molar transformations, we considered the IgA-monomer in serum and IgA-dimer in saliva, as circulatory IgA is most commonly monomeric lacking the SC, whereas IgA found at mucosal surfaces and in mucosal secretions is mostly in the dimeric form (29).

### Robust anti-RBD IgA response in saliva of vaccinees

Given the substantial IgA amounts in serum elicited by the vaccine and the well-established role of secreted IgA in providing mucosal immunity, we asked whether RBD-reactive IgA can be detected in resting saliva of vaccinees. Saliva samples (N = 82) were obtained from 33 participants, aged 20–75 (see **Tables S5** and **S8**, cohort details).

**Figure 3:**
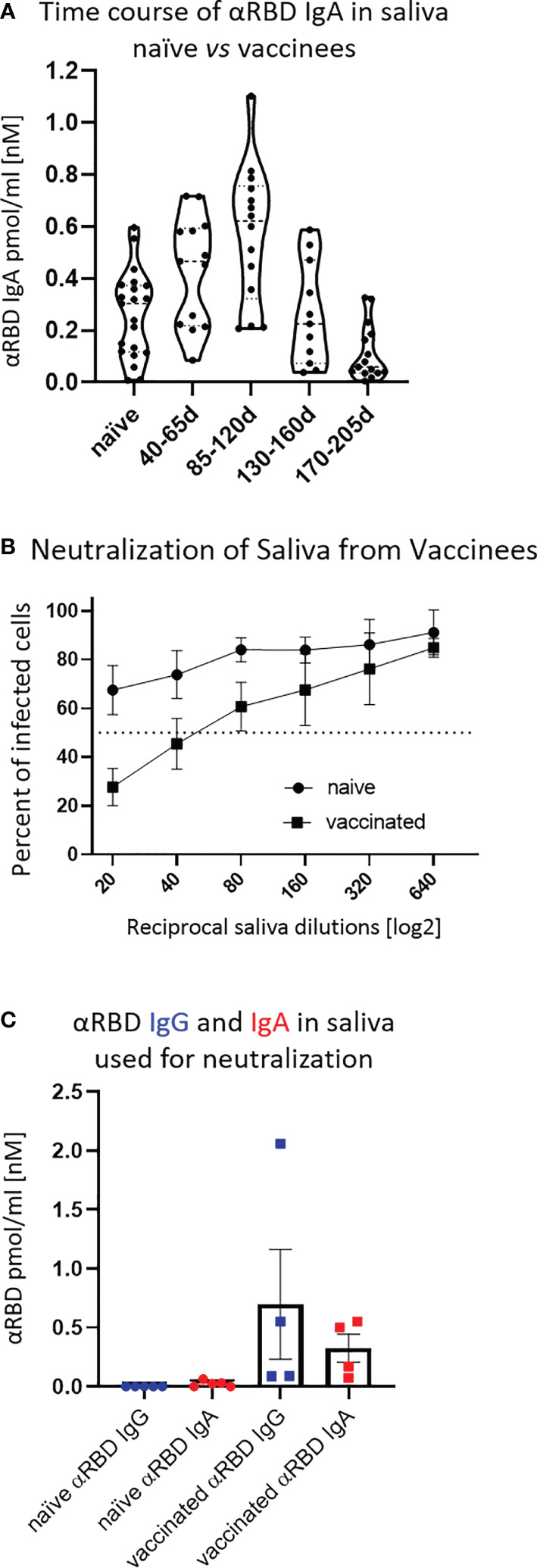
Detection of anti-RBD IgA in resting saliva of BNT162b2 vaccinees and characterization of its neutralizing potential. **(A)** Longitudinal assessment of molar quantities of anti-RBD IgA in saliva of vaccinees compared with naïve individuals is presented in picomole per ml and time-categorized as indicated. The molar expression in saliva is corrected to bi-valent for the comparison to circulatory immunoglobulins. **(B)** Saliva neutralization assessed by SARS-CoV-2 spike-pseudotyped VSV-GFP-ΔG reporter assay on Vero-E6 cells. Neutralization is expressed as a percentage of pseudovirus-infected green cells without incubation with saliva (total infection = 100%). Percentage of independently measured neutralization by five naïve individuals vs. four vaccinees are plotted as a function of the reciprocal values of sera dilutions displayed on a log2 scale, as indicated. Each neutralization curve was tested in three biological replicates. Standard deviation represents difference between individuals in each group. The NT50 of vaccinees saliva is achieved on average at the dilution of ∼1:60, extrapolated by cross-section with the dashed line. The specific neutralization NT50 value is reached at dilution of ∼1:20 and represents “vaccine-added” neutralization, corrected to the basal innate neutralization of naïve individuals, that is probably the consequence of innate proteolytic and mucus (lectin) presence in naïve saliva. **(C)** The values of anti-RBD IgA and IgG in picomole per ml of five naïve saliva samples and four saliva samples of vaccinees, used in neutralization assay described in **(B)** are shown.

First, we confirmed that the vast majority of total immunoglobulins in saliva detected by our quantitative ELISA were of the IgA isotype, in agreement with the well-characterized humoral repertoire of the salivary milieu (**Figure S3A**). Next, we turned to quantify the RBD-specific IgA in saliva samples collected at different time points after vaccination, as indicated (**Figure 3A**). Anti-RBD salivary IgA response was rather variable between individuals, akin to its variability in serum. There was a time-dependent increase in anti-RBD IgA, peaking at about 3–4 months after vaccination and then decreasing steeply at about 5 months after vaccination, dropping to the levels of naïve individuals by the end of 6 months. Notably, the duration of mucosal anti-RBD IgA was significantly extended when compared with circulatory IgA, suggesting involvement of certain aspects of mucosal immunological memory (see Discussion).

The presence of anti-RBD immunoglobulins in saliva is rather encouraging, although the question remains as to its ability to prevent virus entry. Using the VSV–GFP–SARS-CoV-2–Spike pseudotype neutralization assay, a strong concentration-dependent neutralizing activity of saliva from vaccinees was discovered (NT50 ∼ 1:60) (**Figure 3B**, squares, four vaccinee samples). This value is significantly higher than the basal background neutralizing activity of saliva from naïve individuals (**Figure 3B**, circles). The background neutralizing activity of saliva from naïve individuals may stem from basal innate antiviral properties of naïve saliva (e.g., proteolytic digestion and lectin properties). For the sake of sterility while performing the neutralization assay, we used pre-diluted saliva samples that were cleared by centrifugation (12,000g, 5 min) and subsequently size-filtered (0.22 µm) (see **Figure 3C** for the ELISA of clarified saliva samples used in **Figure 3B**). The solubility of IgA molecules in saliva is often a matter of concern due to the viscous-colloid, mucus state. We confirmed quantitative recovery of solubilized saliva IgA by comparing pre- and post-centrifugation and filtration samples by quantitative ELISA (**Figure S3B** and **Tables S6**, Part I and Part II).

### Depletion of IgA from saliva samples of vaccinees abrogates neutralization activity

**Figure 4:**
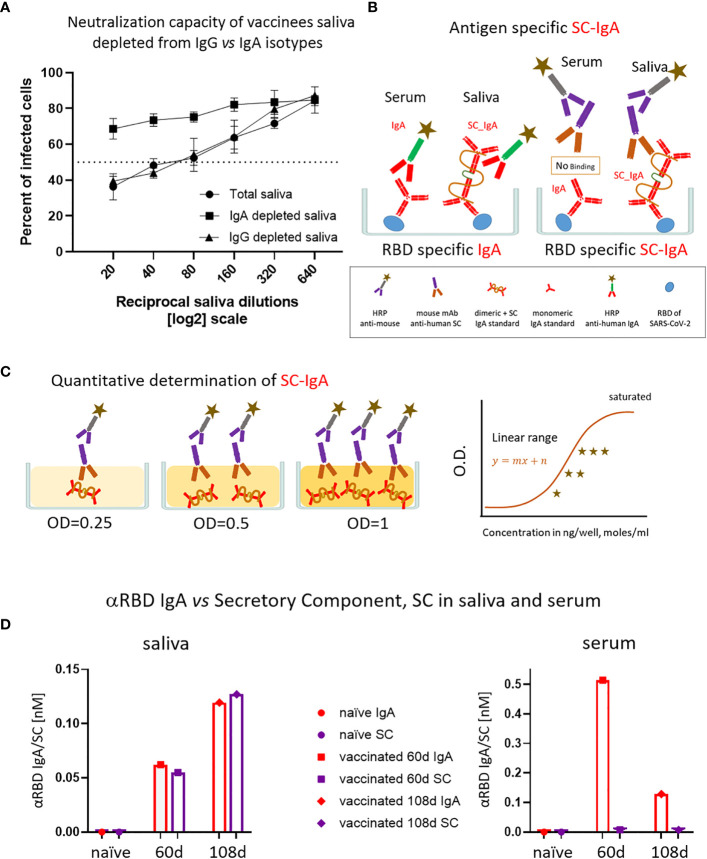
The association of salivary anti-RBD IgA with the secretory component governs the prominent neutralization activity in vaccinees. **(A)** Depletion of IgA from saliva samples of vaccinees completely abrogates the specific neutralization activity of vaccinees saliva. Saliva neutralization was assessed by SARS-CoV-2 spike-pseudotyped VSV-GFP-ΔG reporter assay on Vero-E6 cells. The magnitude of neutralization is expressed as a percentage of pseudovirus-infected green cells without incubation with saliva (total infection = 100%). Percentage of measured neutralization by saliva pool of five vaccinees is plotted as a function of the reciprocal values of the saliva dilutions displayed on a log2 scale. The NT50 of saliva pool is reached on average at the dilution of ∼1:60 (extrapolated by the cross-section with the dashed line). Depletion of IgA results in abrogation of vaccine-induced neutralization activity (squares), whereas IgG-depleted saliva pool coincides with the non-depleted pool (triangles vs. circles). Depletion is achieved using anti-IgA and anti-IgG–specific magnetic beads. Results of three experimental repeats are represented. Analyses of completeness of isotype depletion and of its specificity are presented in **Figure S4A**. **(B)** Schematic outline of the detection of anti-RBD IgA in serum and SC-associated anti-RBD IgA in saliva samples. Illustrated are the expected differences between the circulatory monomeric IgA and the salivary mucosal dimeric/polymeric IgA, covalently bridged by J-chain and associated with pIgR. Left panel shows non-discriminative detection of both isoforms by anti-IgA secondary HRP-conjugate. Right panel shows the selective quantitative determination of dimeric/polymeric secretory IgA in saliva, but not in the serum, using anti-SC mouse monoclonal Ab, followed by anti-mouse secondary detection. **(C)** Molar quantification of dimeric secretory IgA. We introduce as reference standard using commercial secretory dimeric IgA purified from human colostrum to transform the OD values to their molar equivalents. We assume average molecular weight (MW) of dimeric secretory IgA = 424 g/mole. We considered IgA in its dimeric form because it is mostly found in that form at the mucosal surfaces and in mucosal secretions. **(D)** Analysis of dimeric anti-RBD SC-IgA in saliva (upper panel) vs. monomeric anti-RBD IgA in serum (lower panel) after vaccination, measured by quantitative ELISA. The molar expression in saliva is corrected to bi-valence to simplify the comparison to circulatory immunoglobulins.

Given the IgA prevalence in saliva, we next evaluated its functional contribution to neutralization using a limited subset of the available samples (**Figure 4A**). We generated a pool of five saliva samples from vaccinees and subsequently depleted either the IgA or IgG molecules (**Figure S4A**). Depletion of IgA, but not IgG, resulted in the loss of neutralization (**Figure 4A**). This result confirms that the strong neutralizing activity of vaccinees is attributed to IgA.

Salivary IgA, similar to all mucosal IgA forms, is produced in a polymeric form (dimeric-tetravalent or higher-order oligomers), as opposed to predominantly bivalent IgA and IgG monomers found in circulation. To verify whether this is the case in the saliva samples of vaccinees, we employed an anti-SC quantitative ELISA, measuring molar values of total and anti-RBD secretory dimeric IgA (experimental flow is depicted in **Figures 4B, C**, see details in **Supplementary Materials**). We compared a pool of four saliva samples from naïve individuals to the two pools collected from vaccinees at 2 and 3 months after vaccination. In parallel, we analyzed the corresponding serological samples. **Figure 4D** demonstrates that anti-SC reveals RBD-targeting reactivity solely in the saliva of vaccinees and not in their sera. At 60 days after vaccination overall anti-RBD IgA reactivity dominated in serum, whereas the SC-associated anti-RBD form predominated in saliva, in accordance with the strikingly eminent specific neutralization potency of salivary IgA (**Figures 3C**, **4D**). In contrast to a sharp decline of anti-RBD IgA in serum at day 108 (3.5 months) after vaccination, the salivary IgA associated with the SC were mounting, in line with data shown in **Figures 1B**, **3A**. We conclude that anti-RBD IgA in saliva of vaccinees originates from a *bona fide* transcytotic secretory pathway, validating its polymeric nature (**Figure 4D**).

### Strong neutralizing activity of salivary polymeric SC-IgA vs. serum IgG and IgA

To evaluate the specific neutralization potency of the anti-RBD immunoglobulins, we normalized their relative NT50 values to their actual (nM) concentration in the respective fluids (**Table 1**). This pointed to a two orders of magnitude advantage of saliva anti-RBD IgA (NT50 ∼ 0.02–0.05nM) vs. serum anti-RBD IgG (NT50 ∼ 1 nM). We hypothesize that the remarkable neutralization potency of polymeric salivary IgA (relative to monomeric serum IgG) may stem from a combination of (i) the increased avidity of multivalent binding and (ii) a geometrical fit between dimeric/oligomeric IgA and the SARS-CoV-2 spike protein trimer as presented on the surface of virions.

**Table 1:** Neutralizing activities of Serum vs. Saliva immunoglobulin isotypes.

	Serum Abs Concentration [nM]	Serum NT50 [nM] Isotype Specific	Saliva Abs Concentration [nM]	Saliva NT50 [nM] Isotype Specific
αRBD IgG	300.00	~1nM	0.4	ND
αRBD IgA	55	ND (>1nM)	0.65	0.02-0.05nM

Quantitative, molar measurements of anti-RBD immunoglobulin content in saliva and serum allow the evaluation of the specific neutralizing activity expressed as NT50 per (nM) of anti-RBD IgA and IgG per in saliva and serum, respectively. To calculate these values, we normalized NT50 expressed in dilutions of serum and saliva (**Figures 1**, **3**) to the molar concentration in saliva and serum, respectively (nM). NT50 value for salivary IgA was calculated on the basis of average NT50 dilution of ∼1:20, upon normalization to basal inhibitory activity of naïve saliva (see **Figure 3B**). NT50 value for salivary anti-RBD IgA was calculated on the basis of average NT50 dilution of ∼1:20; NT50 value for serum anti-RBD IgG was calculated on the basis of average dilution of ∼1:300. The plausible mechanisms behind such stark, two orders of magnitude difference in NT50 between salivary and serum immunoglobulins are addressed in the “Thought experiment” described below and summarized in the model presented in **Figure S6**.

Whereas the avidity components of multivalent binding and neutralization are well studied (62) in viral infections, the subject of complementarity between a virion lattice and the immunoglobulin isotype is less explored. Simplified views of the molecular dimensions of SARS-CoV-2 spike and of the studied immunoglobulin isotypes are presented in **Figure S6** (molecules are drawn schematically with respect to their proportional scale). Because the surface glycoprotein lattice is sparse (e.g., majority of trimeric spike vertices are 20–25 nm apart) (63, 64), circulatory IgGs and IgAs, being mostly monomeric, might bind to only a single glycoprotein spike, restricted by their Fab arm spread of (10–14 nm). In contrast, dimeric/oligomeric SC-IgA can concomitantly capture at least two glycoprotein spikes due to its > 25-nm longitudinal extension, thereby more efficiently covering—”mantling”—the virion surface. In this view, the “mantling efficiencies” of polymeric secretory IgA are reminiscent of a mythical warrior (*Sanskrit* “*Virabhadra* -}**{**“) and might by far outperform the restricted capabilities of IgG (**Figures S6B, C**, left).

**Figure S6C** illustrates the whole-virion perspective and outlines an additional layer of plausible protection, whereby SC-IgA induced inter-particle oligomerization is promoted by the extended and elastic tetravalent branches. Such a situation, fortified by a plethora of polyclonal species, may appear as highly prominent *in vivo*. In oral immunity, the aggregation of exogenous particles is known to promote mechanical clearance of invaders from mucosal surfaces (65, 66). The consequences might become even more significant in the case of a higher degree of multimerization (30, 67–69). Such an effect was recently reconfirmed for human intranasal IgAs. Intranasal vaccination with *Influenza* virus in humans revealed extreme potency and “neutralization-breadth” toward variant strains. This powerful protection was attributed to nasopharyngeal SC-IgA with the elevating superiority of the multimeric states: dimers, trimers, tetramers, and even higher-order oligomers (67, 70).

Our findings, in conjunction with the “*GedankenExperiment*” (**Figures S6B, C**) of interaction modalities between surface SARS-CoV-2 lattice and mucosal dimeric/oligomeric IgA vs. predominantly monomeric IgG and IgA in blood circulation, highlight the importance of implementing lattice design to improve the spatial surface-mimicry in the next-generation subunit vaccines. In this respect, the mRNA-based vaccine may have had an unexpected benefit by enabling the host cell to present the natural arrangement of SARS-CoV-2 spike membranal lattice upon its expression.

Overall, our results demonstrate that the BNT162b2 vaccine induces a 5-month transient accrual of salivary anti-RBD IgA, extending beyond the time frame of detectable circulatory IgA, putting forward a basis for the establishment of mucosal memory. We suggest that the polymeric origin of the salivary IgA molecules may be responsible for the high specific neutralizing activity found in the BNT162b2 vaccinees’ saliva, compared with serum IgG. Whether salivary anti-RBD IgA represents a more general nasopharyngeal humoral component of mucosal protection needs to be further investigated.

## Discussion

Our study reveals a mucosal component resulting from the intramuscular administration of an mRNA vaccine. We show that saliva of vaccinees contains transitory anti-RBD polymeric secretory IgA (**Figures 3A**, **4D**) with strong neutralizing activity (**Figure 3B**; **Table 1**), possibly explained by its polyvalent nature. We show that this polyvalent IgA is the main mediator of neutralization activity in the vaccinees’ saliva, remaining unchanged following IgG depletion. Accordingly, vaccine-induced neutralization was abolished by depleting salivary IgA. In contrast, IgG was the predominant neutralizing isotype in serum, as its removal resulted in a loss of neutralization. Intriguingly, and contrary to the situation in saliva, residual serum IgA was devoid of measurable neutralization activity, despite its significantly higher concentration—about 30-fold higher IgA content in serum vs. saliva (when valence differences are accounted, see Materials and Methods).

The unique feature of mucosal IgA is its association with the SC that mediates trans-epithelial delivery of polymeric immunoglobulins and extends their lifespan in the highly hydrolytic mucosal environment. The functional and mechanistic impacts of such associations in terms of avidity and stereochemical properties are discussed below. Epitopic repertoires of salivary and serum IgA may also differ due to affinity maturation driven somatic hypermutations of nasopharynx-associated lymphoid tissue (NALT) resident B-cell clones. Nevertheless, our NT50 molar measurements, intrinsically normalized to the binding reactivity values, assessed in ELISA, argue in favor of superior neutralization by salivary IgA due to its polymeric origin.

Anti-RBD IgA remained present in saliva for an extended period of time after vaccination (it peaked at 2–4 months and vanished only 5–6 months after vaccination), significantly outliving serum anti-RBD IgA (**Figure 4D**). While analysis of the sustained immunological memory mediated by NALT and broncho-alveolar associated lymphoid tissues (BALT) is pending, one might wonder about the possible impacts of systemic or even locally applied mucosal boosts, e.g (28, 71, 72). Because the presence of mucosal IgA and functional importance in recovered individuals are now well characterized (38, 39), it might be interesting to further monitor the mucosal-effect of post-recovery vaccine boost, e.g (41, 73). The dynamic epidemiological reality, however, is often more complex, given the antigenic diversity of the rapidly emerging SARS-CoV-2 variants. In this view, adaptation of mucosal boosts to emerging variants may be considered in the future, e.g (74, 75).

It is not yet clear whether BNT162b2 mRNA vaccination provides temporary sterilizing immunity in addition to its proven capacity to ward off severe disease. “Sterilizing immunity” is crucial to interfere with the spread of SARS-CoV-2 and reduce the emergence of new variants, although this may be limited by emerging antigenic escape properties of the variants and by possible virus re-introductions through reverse zoonosis. Of note, the time frame of the delta-variant infection wave in Israel, where the majority of population was vaccinated by BNT162b2 approximately 5–6 months prior to the wave spread (76), coincidentally correlates with our findings of a drop in salivary IgA after vaccination (**Figure 3A**). Whether waning immunity at the population level had a causative relationship with the drop of systemic anti-RBD IgG or mucosal anti-RBD IgA or with the immune escape of the emerging variants and their relative involvements remains to be investigated. Mucosal IgA is indeed often transient even when induced by natural mucosal invaders; however, the immunological memory initiated at the *lamina propria* may reside in place, or in secondary lymphoid organs, ensuring an inducible defense response. It still remains unknown whether such mucosal humoral memory endures in vaccinees or in recovered patients and, if so, to what extent it reacts to avert the spread of infection. In this vein, recent studies in mice demonstrate the ability of adenovirus vectored intranasal boosts to achieve complete SARS-CoV-2 protection by (1) inducing high level mucosal neutralizing IgA and (2) stimulating NALT resident memory T cells, when administered after primary mRNA or plasmid DNA intramuscular vaccination (71).

The ideal vaccine is aimed to provide a perfect mimicry of the natural infection route and, as such, would train the immune response to situate its guards “*en place*”. The well-studied poliovirus case with the known difference between inactivated polio vaccine and oral polio vaccine exemplifies the importance of such mimicry for providing sterilizing immunity (77, 78). Many more studies also demonstrate that the mucosal route of vaccination provides such a beneficial protection against respiratory and digestive-tract virus and bacterial infections, including influenza and rotavirus and even SARS-CoV-2 and its emerging variants, e.g (5, 27, 67, 71, 79–86). However, formulating an immunogenic, broad, and safe “subunit” or “inactivated” mucosal viral vaccine, capable of eliciting long-term efficient and balanced mucosal-plus-systemic protections, remains challenging (87). Several non-mucosal vaccines were shown to induce the mucosal component of protection, e.g (27, 88). Whereas intramuscular DNA vaccines widely studied in animal models are known to possess such capabilities (89, 90), Whereas intramuscular DNA vaccines widely studied in animal models are known to possess such capabilities, instramuscular lipid mRNA formulations in human settings have not yet been widely characterized, particularly with respect to its mucosal aspects and its functional protection. Although not tested here, one could assume that the elicited mucosal humoral immunity might not be restricted to saliva and may afford broader mucosal protection, extending to (1) the nasopharyngeal niche elicited by NALT (2), the lower respiratory Broncho-alveolar mucosa brought about by BALT, and even (3) the gastrointestinal tract mediated by GALT. Additional studies will be needed to further explore the immunoglobulin composition and potency at these mucosal sites.

Recent reports indicate the presence of anti-SARS-CoV-2 IgA in breast milk of BNT162b2-vaccinated women (91, 92). Support for the presence of mucosal IgA following mRNA SARS-CoV-2 vaccination comes from several additional recent studies, e.g (73, 80, 93–95). For instance, Chan et al. have compared two different SARS-CoV-2 parenteral vaccine platforms approved for emergency use in Hong Kong for their ability to induce neutralizing IgG/IgA in serum vs. nasal epithelial lining fluid (NELF): CoronaVac (inactivated virus vaccine) and Comirnaty (mRNA vaccine) (93). Intriguingly, Comirnaty induced an anti-spike neutralizing IgA response detected in NELF, whereas a similar response was not observed in CoronaVac vaccinees, highlighting the mucosal capabilities of mRNA-based vs. inactivated vaccine (93).

Importantly, however, several recent studies comparing naïve and post-recovery SARS-CoV-2 mRNA vaccination in NELF and in bronchoalveolar lavage indicate that mRNA vaccination is significantly more efficient at boosting than at priming mucosal immunity, e.g (73, 80), and suggest cross-protection against emerging variants of concern (96, 97). This aspect relates to earlier studies which report instances whereby, whereby systemic immunization was capable of boosting mucosal SC-IgA in individuals who had previously encountered an antigen by a mucosal route, either by natural infection or by primary mucosal vaccination, e.g (27, 28).

Despite the importance of IgA for protection against pathogens, a certain fraction of the human population is characterized by IgA deficiency (98, 99). Only 10%–15% of IgA deficient individuals are susceptible to recurrent sino-pulmonary and gastrointestinal infections/disorders, whereas the vast majority remain asymptomatic and are often accidentally identified among healthy blood donors. In many cases, IgM appears to compensate the deficiency by replacing IgA at mucosa, as it reacts with pIgR and can be transcytosed to mucosal surfaces (98). Whether mRNA vaccines boost mucosal IgM as well in such instances of IgA deficiencies remains to be explored.

The mechanism of eliciting the mucosal humoral component by an mRNA vaccine remains mysterious, but the presence of the SC points to the transcytotic origin of the polymeric isoform in saliva. Such SC-IgA is most likely generated by dedicated B cells situated at the *lamina propria*. These B cells could either originate from local stimulations, or alternatively, could be re-targeted for homing from the bone marrow or secondary lymphatic organs (e.g., from local NALT lymph nodes, from spleen, or from distal mucosal sites, such as Peyer’s patches) (24, 27, 100, 101).

Local antigenic stimulation may originate from either the lymphatic drain of the spike protein produced at the site of injection or from the trafficking to the *lamina propria* of the mRNA itself, being subsequently expressed by either NALT, GALT, or the epithelial cells. Analyzing local MHC-I vs. MHC-II T-cell responses could help to distinguish between the two scenarios (102, 103), although potential cross-presentation by dendritic cells may complicate the analysis (104). Such a hypothetical delivery of mRNA or of the expressed spike-antigen to the mucosa and its potential immunological impact are intriguing and worth a detailed investigation. Plausible routes could involve (i) a lymphatic drain facilitated by liposome-directed targeting, (ii) a natural exosome-mediated delivery route to distal anatomical sites, or (iii) migration of antigen presenting cells from the site of expression to secondary lymphoid organs. Mechanisms behind such putative scenarios have been previously suggested (105–110).

Thus, the origin of salivary SC-IgA in systemically vaccinated individuals remains to be explored. Does it originate from the polymeric IgA-producing plasma cells in systemic lymphoid tissues or from the bone marrow, or is it produced locally in the parotid, submandibular, sublingual, or minor salivary glands of the oral cavity? Of note, circulatory immunoglobulins, both monomeric and polymeric IgA, IgG or IgM, were reported not to effectively traverse into external secretions, including the saliva (24, 27, 101, 111)

Whether the pre-existing immunological memory at mucosal sites to former instances of respiratory human common cold coronaviruses (e.g., OC43, NL63, HKU1, and 229E) is stimulated by intramuscular mRNA boost, panning cross-reactive B cells, remains to be seen (56, 57).

The approach that we introduce here for the evaluation of anti-SARS-CoV-2 humoral response relies on molar units of antigen specific and of total immunoglobulins. Such molar expressions are well-adopted in clinical diagnosis of autoimmune diseases and provide universal international evaluation and decision-making in patients’ care (112, 113). In line with this view, several recent studies have introduced universal unit measurements for analysis of circulatory immunoglobulin responses against SARS-CoV-2 antigens using “spike-in” and reference approaches, e.g (56, 60). Beyond the obvious benefits of such universality for surveillance and comparative research, our work demonstrates the instrumental importance of absolute units and standardization for mechanistic understanding of functional neutralization.

Comparison of neutralizing activities in serum and saliva upon BNT162b2 vaccination suggests the first *in vivo* evidence of augmented specific neutralization of polymeric IgA. At first glance, “the multivalent state *per se*” is an obvious explanation, following the orthodox proximity-based statistical models of association-dissociation shift—the “avidity” component (114). The valence influence is often more prominent for weak affinities, in agreement with the expected shift in the association-dissociation probabilities. Such an avidity component suggests an intriguing possible benefit in the protection toward emerging variants even at the expense of drop in affinities. This classical view is well-studied and has multiple experimental confirmations, such as comparing kinetic binding and neutralization properties of monovalent Fabs vs. bivalent IgGs and bivalent IgAs vs. tetravalent IgAs, e.g (67). Nonetheless, we would like to emphasize that the geometric match between the glycoprotein matrix on the virion surface and the antibody architecture may significantly impact the neutralization efficiency, given the molecular dimensions of the spike protein. Several recent structural studies have employed cryoelectron tomography to analyze spatial stereochemistry of the authentic SARS-CoV-2 particles (63, 64). Remarkably, the reported center-to-center distances between the trimeric spike foci on the virion surface peak at approximately 20–30 nm, matching the 25-nm longitudinal axis of the dimeric IgA (**Figure S6B**). Higher-order secretory IgA oligomers would have greater reach and thus an even stronger influence, e.g (69, 70).

Several recent studies reported the presence of powerful mucosal IgA in post–COVID-19 patients, e.g (38, 39, 80). Furthermore, Wang et al. have recently shown that, in a recombinant setup, monoclonal IgAs subcloned from circulatory PBMCs of recovered COVID-19 patients exhibit elevated neutralizing potential upon co-expression with the dimer-forming joining chain (37). Recent biotechnological studies have established *ex vivo* systems for the efficient recombinant production of dimeric IgA containing the SC, e.g (115).

Whereas the current study illuminates the mucosal aspects of BNT162b2 mRNA vaccine, many additional vaccines are already implemented. Although systemic immunity of these vaccines is often thoroughly compared, e.g (116), their mucosal components are much less explored, e.g (93, 117). Many more of different introduction routes are in clinical trails and development, including mucosal administration, holding a promise of eliciting long-term, broad, and potentially sterilizing immunity, e.g (71, 72, 118–123).

In conclusion, our data reveal the existence of spike-targeting temporary mucosal secretory IgA in saliva of BNT162b2 vaccinees and describe their specific neutralization potency determined on a limited cohort. Our approach of molar quantification of SARS-CoV-2 immunoglobulins in various body fluids may have practical implications for basic research and for the accurate assessment of humoral immunity in diagnostics and in epidemiological surveillance studies. Surveying salivary IgA is non-invasive and easily accessible and as such may be beneficial in the search for correlates of protection. 

## Materials and methods

### Cell lines

Vero E6 and HEK293T cell lines were obtained from the American Type Culture Collection. Vero E6 and 293T cells were grown in Dulbecco's Modified Eagle's Medium (DMEM|) medium supplemented with 10% (v/v) fetal bovine serum, penicillin (100 IU/ml), and streptomycin sulfate (100 mg/ml), and the cells were grown in 5% CO_2_ and 95% air. Cells were passaged at 80% confluence and seeded as indicated for the individual assays. Proteins were produced in Expi293 or ExpiCHO that were obtained from Thermo Fisher and grown according to the manufacturer’s instructions.

### Clinical cohort and sample collection

From December 2020 to May 2021 we enrolled participants at Hadassah-Hebrew University Medical Center without previous documented COVID-19 to participate in our study. Eligible participants were both male and female adults prior to or after receiving the BNT162b2 vaccine. The vaccine was provided as part of an ongoing national vaccination campaign. This study was part of an ongoing study and was reviewed by the Institutional Review Board (0278-18-HMO). All the participants provided written informed consent. Serum samples were obtained from each participant; saliva samples were obtained from a portion of the participants (saliva cohort). Whole saliva without stimulation of salivary secretion was collected into 50-ml sterile centrifuge tube (CentriStar Cap, Corning). Collected saliva was immediately stored on ice for up to 30 min after collection and subsequently frozen at −20°C for long-term storage. Saliva samples were subsequently thawed on ice and processed as described below for ELISA, IgG or IgA depletion, or neutralization assays. Multiple serum samples were obtained from some of our participants to investigate the kinetics of immunoglobulins response to the vaccine (longitudinal cohort). These samples were collected in the following time frames indicated in the cohort tables (**Supplementary Materials**) starting from day 0 until day 180 after the first vaccine. [P] refers to the number of cohort participants, whereas [N] refers to the number of samples. Therefore, [N] is more than [P] because there can be many serial samples from one participant. See integrated cohort in **Tables S7**, **S8** for further details.

The vaccinee cohort is mostly based on medical students and personnel volunteers, who were routinely screened for SARS-CoV-2 either by PCR or antigen tests during the outbreak. The volunteers were interviewed to confirm the absence of previous diagnosed disease, however, to exclude asymptomatic cases or other types of undiagnosed cases, we included a full-length nucleocapsid (N) protein antigen seropositivity ELISA assay (**Tables S7**, **S8**). We developed this assay in the laboratory as an additional criterion to identify cohort subjects suspected for undiagnosed previous SARS-CoV-2 infection and to distinguish those from participants that were only vaccinated. N-protein is not a part of BNT162b2 vaccine, which is based only on spike-encoding mRNA. To validate the anti-N ELISA method and to determine the threshold of seropositivity, we relied on pre-COVID-19 samples and clinically validated recovered samples. People that were identified with N ELISA values as suspected but not confirmed undiagnosed SARS-CoV-2 by anti-N, IgG seropositivity are marked with (&) in the integrated cohort in **Tables S7**, **S8**. None of these people were used for our depletion experiments.

Convalescent samples were collected in the period between 3 and 10 weeks after recovery. Recovery is defined by the clinical definition in Israel at time of sample collection. Convalescent plasma and pre-COVID-19 era serum samples were used as a reference for the serum studies; saliva from unvaccinated persons was used as references for saliva studies. The Institutional Review Board approval number for the study and analysis of convalescent samples in whom SARS-CoV-2 infection was diagnosed by RT-PCR at the Hadassah Clinical Virology Laboratory is 0235-20-HMO. The pre-COVID-19 controls were randomly retrieved from the blood-bank serum samples that had been routinely tested at the clinical Virology laboratory between 06-09/2019. The samples were kept at −80°C (sera) and −20°C (saliva). They were heat-inactivated and filtered prior to ELISA or neutralization assays.

### Constructs and plasmids

The following plasmids were used for VSV pseudovirus production: pVSV-ΔG-GFP, pCAGGS-G, or pBS-N-Tϕ, pBS-P-Tϕ, pBS-L-Tϕ, and pBS-G (124).

The plasmids for expression of the receptor-binding domain of SARS-CoV2 spike (RBD) and full-length spike [SARS-CoV-2 S (Δ19 aa)] were cloned into the pcDNA3.4 backbone (Thermo). The sequences were amplified from SARS spike synBio (SARS-CoV-2 (2019-nCoV) plasmid using specific primers. The amplified PCR fragments were subsequently cloned using Gibson assembly reaction into pcDNA3.4 backbone modified to include C-terminal Strep Tag-II (IBA). The sequence of the cloned plasmids was verified using Sanger sequencing. All plasmids were amplified under ampicillin selection in Top10 cells (Invitrogen) and purified by NucleoBond Xtra Midi EF kit (MACHEREY-NAGEL).

### Protein expression and characterization

#### Receptor-binding domain protein

RBD of SARS-CoV-2 spike was expressed in mammalian expression system using transient transfection (Expi293 or ExpiCHO) as a secretory protein with C-terminal sreptag and subsequently purified using streptactin affinity chromatography on 5-ml Strep-Tactin^®^ Superflow^®^ high capacity cartridge (IBA), followed by preparative size exclusion chromatography using HiLoad 26/600 Superdex 200 pg column on the Fast Protein Liquid Chromatography ÄKTA pure system. The purity and molecular weight of the antigen were verified using Laemmli discontinuous SDS-PAGE, 12% (sodium dodecyl sulfate–polyacrylamide gel electrophoresis) (125), samples were denatured and reduced prior to run by heating to 95°C for 5 min in Laemmli sample buffer containing freshly added 100 mM DTT. To determine molecular weight and degree of glycoconjugation of the antigen in native state in solution, samples were analyzed by size-exclusion chromatography multiple-angle laser scattering (SEC-MALS). SEC-MALS was performed on HPLC (SHIMADZU DGU-20A, UFLC), equipped with autosampler, UV, and fluorescence detectors (SIL-20AC HT and LC20AD) using Superdex 200 Increase 10/300 GL column. Samples were run in 20 mM Tris and NaCl 150 mM (pH 7.5) at a flow rate of 0.4 ml/min. These samples passed in-line, through a Wyatt DAWN Heleos II EOS 18-angle laser photometer detector coupled to a Wyatt Optilab TrEX differential refractive index detector. Data were later analyzed to determine molecular weight in solution and for glycoconjugate analysis (**Supplementary Materials** and **Figure S1B**) using Astra 7 software (Wyatt Technology Corp).

#### Nucleocapsid protein

N-protein of SARS-CoV-2 was expressed in Escherichia coli T7 express (New England Biolabs) and grown in 2xYT medium. Cultures were inoculated using 1% (v/v) overnight saturated cultures and were grown at 37°C to an OD600 of 0.5–0.8. Protein was induced at 16°C overnight by addition of 300 μM isopropyl-β-d-thio-galactoside. Cells were harvested by centrifugation (8,000g, 15 min) and stored at −80°C for later use. N-protein was purified by resuspending cell pellets in lysis buffer [50 mM Na-phosphate (pH 8.0), 500 mM NaCl, 10 mM imidazole, 5 mM BME, and 3 M urea] after adding 1 mM phenylmethylsulfonyl fluoride. Cells were disrupted using a Microfluidizer (Microfluidics), and the lysate was centrifuged at 20,000g for 1 h to remove cell debris. The lysate was subjected to immobilized metal affinity chromatography (IMAC) using 5-ml His-Trap columns (GE Healthcare). The column was washed with decreasing concentrations of urea to a final buffer of lysis buffer without urea. Finally, protein was eluted using a linear imidazole gradient of 15–300 mM in 30 column volumes. Fractions containing purified protein were pooled, and TEV protease was added in a ratio of 1:100 and dialyzed overnight at 4°C against lysis buffer without imidazole or urea. Cleaved protein was then subjected to a second round of IMAC and the cleaved protein was collected from the flow-through and was loaded on Superdex 200 gel filtration column (GE Healthcare) in 20 mM Tris (pH 7.5), 500 mM NaCl, and 2 mM DTT. The elution peak was concentrated and flash-frozen in liquid N_2_.

### Direct enzyme-linked immunosorbent assay

Antigen (RBD) was coated onto MAXISORB 96-well plates in antigen dilution buffer [20 mM Tris (pH 7.5) and 50mM NaCl]. Following overnight (ON) incubation at 4°C, plates were rinsed three times with PBS and then blocked with 3% fat ultra high temperature milk:PBS (1:1), 30 min, room temperature. Sera samples and saliva samples (1:1 with PBS) were heat-inactivated (60°C, 30 min), serially diluted in blocking buffer, and then added to the wells and incubated for 45 min at RT. Wells were rinsed (three times with PBS) and incubated with secondary Abs coupled to HRP (anti IgA at 1:2,500 or IgG at 1:5,000) for 30 minutes, rinsed, and then, wells were developed with 3,3′,5,5′-Tetramethylbenzidine substrate. The reaction was stopped with 0.4% H_2_SO_4_ and read at 450 nm in an ELISA reader (Spark, Tecan)

To expand the dynamic range of the assays, the samples (sera or saliva) were always applied as a series of two- or three-fold dilutions. The OD values correlating with the dilutions all fell within the linear OD range (“Normalized OD”). Therefore, all samples are diluted to be within the linear range of the ELISA read-out.

### Sandwich ELISA for total IgG or IgA

MAXISORB 96-well plates were coated ON with secondary anti-IgG or anti-IgA antibodies, blocked as for direct ELISA, and incubated for 30 min with sera and saliva samples, serially diluted in blocking buffer. The wells were then washed (three times with PBS), incubated with secondary Abs coupled to HRP, and developed, as described for the direct ELISA protocol.

### Quantitative ELISA

Pure commercial antibody was measured *via* nanodrop, at 280 nm (ThermoFisher) and then diluted serially to final concentrations of 4.40 ng/100 μl, 1.46 ng/100 μl, 0.48 ng/100 μl, and 0.16 ng/100 μl. ELISA was performed on these four dilutions and then a linear graph was made to calculate the conversion between the OD value of the sample and the corresponding value in ng/100 μl (ng/well). The R^2^ value of this equation was always at least 0.98. This ladder was included in every experiment so that for each sample falling within the linear range of the ladder, the equation could be used to calculate the value in ng/well. Samples with an OD value higher than the least diluted commercial sample or lower than the most diluted commercial sample were excluded from the data set. The ng/well (ng/100μl) value was then multiplied by the dilution factor of that given sample and converted to molar concentration (M) using the molecular weights of 146 kDa for IgG, 150 kDa for IgA, and 424 kDa for dimeric IgA. The fold dilutions were integrated in the quantitative molarity calculations and checked to make sure that the same quantitative value was received for a given sample in at least two consequent dilutions that fell within the detectable range. To further assess the robustness of our calibration curves, we performed additional expanded calibration curves presented in **Figure S5**.

### Selective IgG and IgA depletion

Streptavidin-coated magnetic beads, coated with biotinylated anti-human, isotype-specific antibodies (anti-IgG or anti-IgA), were used to achieve complete and selective depletion of IgG and IgA isotypes from serum and saliva samples, as detailed below. Streptavidin magnetic beads (# NEB-S1420S), a slurry of 4 mg of beads per ml, were washed four times with PBS and then incubated for 45 min with biotinylated capturing antibody, either Biotin-SP AffiniPure Donkey Anti-Human IgG (H+L) (minimal cross-reactivity against bovine, chicken, goat, guinea pig, syrian hamster, horse, mouse, rabbit, rat, and sheep serum proteins, #709-065-149, Jackson ImmunoResearch, PA, USA) or Biotin-SP AffiniPure Goat Anti-Human Serum IgA, α chain specific (#709-065-149, Jackson ImmunoResearch, PA, USA). Biotin-SP anti-human antibodies were diluted 1:10 in PBS and added to beads at a concentration of about 30 μg of antibodies per 1mg of beads. In addition, 12 mg of beads, or 3 ml of slurry, were prepared for each sera depletion (400 μl, serum), whereas 2 mg of beads were prepared for each depletion of saliva (450 μl of saliva sample previously pre-diluted with PBS 1:1). Following incubation, the beads were washed an additional four times with PBS before the addition of the sera or saliva samples. The samples were incubated while rotating at 4°C for 45 min with the beads that were subsequently separated on the magnetic stand. The samples were then checked *via* ELISA to determine whether complete and isotype selective depletion had been achieved. Cross-reactivity was checked *via* ELISA; see **Figure S2D**.

### Preparation of pseudotyped VSV and neutralization assay

VSVΔG-GFP single-round infectious particles were first generated by pseudotyping with VSV-G-envelope expressed *in trans*. P0 generation was produced according to the original Michael Whitt (124) protocol with minor modification, using co-transfection of the five plasmids (pVSV-ΔG-GFP, pBS-N-Tϕ, pBS-P-Tϕ, pBS-L-Tϕ, and pBS-G) into HEK293T cells. Infection of the transfected cells with vaccinia T7 polymerase expressing virus was used to drive cytoplasmic T7-driven transcription and mRNA-capping from the plasmids. P1 generation of VSV-G pseudotyped VSVΔG-GFP particles was generated by transfection of pCAGGS-VSV(G), followed by infection with P0 particles. P2 generation of the SARS-CoV-2 spike-pseudotyped VSVΔG-GFP reporter particles was generated by transfection of HEK293T that were subsequently infected with P1 particles.

### Cell transfection for pseudotyped virus production

HEK293T cells were co-transfected with all five plasmids (see above), carrying defective envelope of VSV plasmid (trans form) and GFP reporter plasmid by using Transporter ™5 transfection reagent (PEI, 40,000 Da, PolySciences), according to the provider’s guidelines. Twelve hours after transfection, the cells were infected with recombinant vaccinia T7 polymerase expressing virus (126) (kindly provided by Prof. Moshe Kotler, The Hebrew University of Jerusalem). P0 pseudo-particles progeny was collected at 48 hours after infection: The supernatant was centrifuged (4,500g, 4°C, 30 min) and filtered (0.1 µm, CA filter) to separate VSV pseudoparticles from vaccinia virus. The complete removal of infectious vaccinia virus was confirmed by end-point titration. The filtrates were aliquoted and stored at −80°C. In the second round of infection to generate P2 pseudo-particles, HEK293T cells were transfected with the plasmid encoding SARS-CoV-2 S (Δ19 aa) by using Transporter™ 5 reagent and 12 hours after transfection infected with VSV-ΔG pseudovirus particles. At 48 hours after transfection, the supernatant was collected and concentrated by Lenti-X™ Concentrator following the manufacturer’s protocol (Clontech, CA). The pellet was resuspended in PBS, aliquoted, and stored at −80°C. Vero E6 cells were seeded in 96-well plate to get 75%–80% confluence and infected with serially diluted SARS-CoV-2 spike (Δ19aa) pseudovirus. The pseudovirus was titrated by counting the green cells 24 hours after infection.

### Neutralization assay

Serum and saliva samples were heat-inactivated (60°C, 30 min) prior to their use in neutralization assays. Saliva samples were pre-diluted 1:1 with PBS before inactivation to avoid coagulation. Next, the samples were diluted in the cell culture growth medium and filtered by using 0.2 μm of cellulose acetate. Vero E6 cells were seeded in 96-well plate and used for neutralization assay at 75%–80% confluency. The sera and saliva samples were serially diluted and subsequently incubated with constant amount SARS-CoV-2 spike-pseudotyped virus for 1 h at 37°C. Next, the mixture was transferred to the monolayer and incubated for 16–24 h. The green fluorescence signal was observed under the microscope at 18–24 hours post-infection . The reduction in amount of green-fluorescent cells due to neutralization was calculated in percentage of un-inhibited control infection. The images were captured from several fields of each well, and the green cells were calculated by using automated image analysis by ImageJ (NIH). The graphs were plotted to get the 50% neutralization titer (NT50) in GraphPad Prism.

## Data availability statement

The original contributions presented in the study are included in the article/**Supplementary Material**. Further inquiries can be directed to the corresponding authors.

## Ethics statement

The studies involving human participants were reviewed and approved by Hadassah Medical Center Institutional Review Board (0278-18-HMO). The Institutional Review Board approval number for the study and analysis of convalescent samples in whom SARS-CoV-2 infection was diagnosed by RT-PCR at the Hadassah Clinical Virology Laboratory is 0235-20-HMO. The patients/participants provided their written informed consent to participate in this study.

## Author contributions

All authors listed have made a substantial, direct, and intellectual contribution to the work, and approved it for publication.
