# Supplementary material for: Intramuscular mRNA BNT162b2 vaccine against SARS-CoV-2 induces neutralizing salivary IgA
Source: Front Immunol. 2023 Jan 30;13:933347. doi: 10.3389/fimmu.2022.933347 (PMC9927016; doi:10.3389/fimmu.2022.933347)
Supplement: Supplementary file 1 [file Presentation_1.pdf]

Supplementary Material

Intramuscular mRNA BNT162b2 vaccine against SARS-CoV-2 induces production of robust neutralizing salivary IgA

Assessment of RBD quality and N-linked glycosylation status and validation of custom ELISA

To evaluate the magnitude and the composition of anti-SARS-CoV-2 acquired humoral immunity, we have developed quantitative ELISA using the recombinant RBD derived from SARS-CoV-2 spike protein (Figure S1A, B). Figure S1A shows single band purity of recombinant RBD. Peptide-*N*-Glycosidase F (PNGase F) sensitivity and the predominant Endoglycosidase H (EndoH) resistance of Asparagine-linked (N-linked) glycans of recombinant RBD, points to appropriate post-translational Golgi-derived glycosylation, as it might occur in the settings of natural infection. Evaluation of RBD by size-exclusion chromatography and multiple-angle laser-light scattering (SEC-MALS) shows monodisperse peak with a molecular weight (MW) of 39.2kDa, corresponding to a monomer in solution (Figure S1B). The RBD glycoconjugate analysis demonstrates that approximately 10% of the MW is attributed to N-linked glycans, highlighting their significance in the RBD surface antigenic properties (Figure S1B). The Pearson correlation coefficient of 0.967 between our quantitative test and the ARCHITECT (Abbott, Illinois, U.S.A.) anti-RBD IgG test, confirmed the reliability of our assay, see Figure S1C, for correlation analysis and Table S3, for cohort details.

Rationale and details of the anti-SC quantitative ELISA

Basolateral B-cells in the *lamina propria* generate J-joined dimers/oligomers of IgA that are then luminally delivered by transcytosis to be secreted at mucosal surfaces. The part of the pIg receptor, cleaved during transcytosis (the secretory component, SC), remains permanently associated with the mucosal IgA and is important for its stabilization. To determine the origin of RBD-specific salivary IgA in vaccinees, we asked whether it contains SC. To this end, we employed anti-SC quantitative ELISA measuring total and anti-RBD secretory SC-IgA (experimental flow is depicted in Figures 4B, C). The molar values were inferred using pure commercially available SC-IgA as a reference standard (Figure 4C).

Figure S1

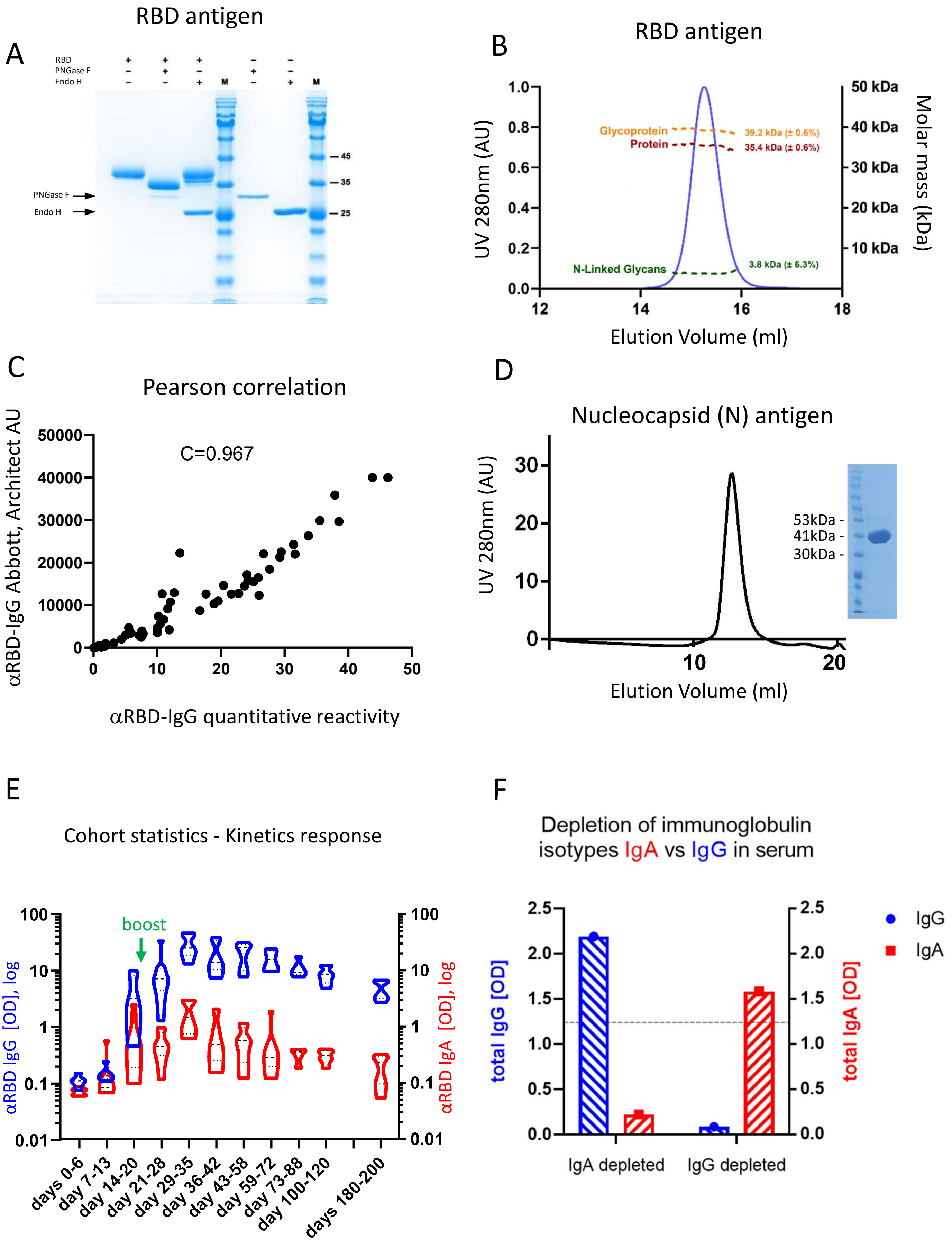
A
RBD antigen
RBD
PNGase F
Endo H
M
45
35
25
PNGase F
Endo H
B
RBD antigen
Glycoprotein
Protein
39.2 kDa (± 0.6%)
35.4 kDa (± 0.6%)
N-Linked Glycans
3.8 kDa (± 6.3%)
UV 280nm (AU)
Molar mass (kDa)
50 kDa
40 kDa
30 kDa
20 kDa
10 kDa
Elution Volume (ml)
C
Pearson correlation
C=0.967
αRBD-IgG Abbott, Architect AU
αRBD-IgG quantitative reactivity
D
Nucleocapsid (N) antigen
UV 280nm (AU)
53kDa -
41kDa -
30kDa -
Elution Volume (ml)
E
Cohort statistics - Kinetics response
boost
αRBD IgG [OD], log
αRBD IgA [OD], log
days 0-6
day 7-13
day 14-20
day 21-28
day 29-35
day 36-42
day 43-58
day 59-72
day 73-88
day 100-120
days 180-200
F
Depletion of immunoglobulin isotypes IgA vs IgG in serum
total IgG [OD]
total IgA [OD]
IgG
IgA
IgA depleted
IgG depleted

Figure S1

Serological assay: characterization of RBD antigen, validation of anti-RBD ELISA and isotype-specific serological depletion. (A) SDS PAGE of recombinant RBD, produced in mammalian cells in a secreted form. Enzymatic removal of N-linked glycans by PNGase treatment results in characteristic electrophoretic mobility shift, while predominant EndoH resistance demonstrates successful ER-to-Golgi transition during the secretory process. The two lanes on the right show the electrophoretic references of the enzymes (arrows). (B) Size-exclusion chromatography and multiple-angle laser-light scattering analysis confirms monodisperse peak in the form of monomer, with the apparent molecular mass in solution of 39.2kDa, glycoconjugate analysis reveals that the contribution of proteinacious core is 35.4kDa and that the N-linked glycans contribute ~ 10 percent of the molecular weight of the mature secreted RBD. (C) Pearson coefficient of 0.967 demonstrates linear correlation of our anti-RBD IgG ELISA to the routine diagnostic kit of Abbott. (D) SDS-PAGE and Size exclusion chromatography of recombinant Nucleocapsid (N) protein of SARS-CoV-2 produced in bacterial cells, see methods for details of expression and purification. (E) Quantitative kinetic profile of anti-RBD IgG (blue) and IgA (red) in serum sampled in the vaccinees cohort, categorized in periods of significance (see Figure 1B, for the uncategorized data presented as a function of days post vaccination). Independent ordinate axes for IgG (left, blue) and IgA (right, red) highlight the restricted, relative nature of the comparison between isotypes in this experiment, as discussed in the text, see also Figure 2 for subsequent developments. Green arrows indicate timing of the second vaccine dose (the boost). (F) Completeness and specificity of IgG depletion from pooled serum samples used for neutralization experiments presented in Figure 1C. Right-side bars (IgG depleted) serum samples were measured by sandwich ELISA for total IgG (blue column on the right) and IgA (red column on the right), see methods for further details. IgA-depleted samples were measured for total IgG (blue column on the left) and for IgA (red column on the left) were used as a reference for completeness and specificity of IgG depletion. IgG and IgA values are represented on separate ordinate axes (indicated), as explained in the main text. Horizontal dashed line indicates saturation level of ELISA measurement. Total immunoglobulins were measured in this experiment in saturated conditions to confirm the completeness of the depletion.

Figure S2

Standard curves for isotype specific ELISA and controls for specificity

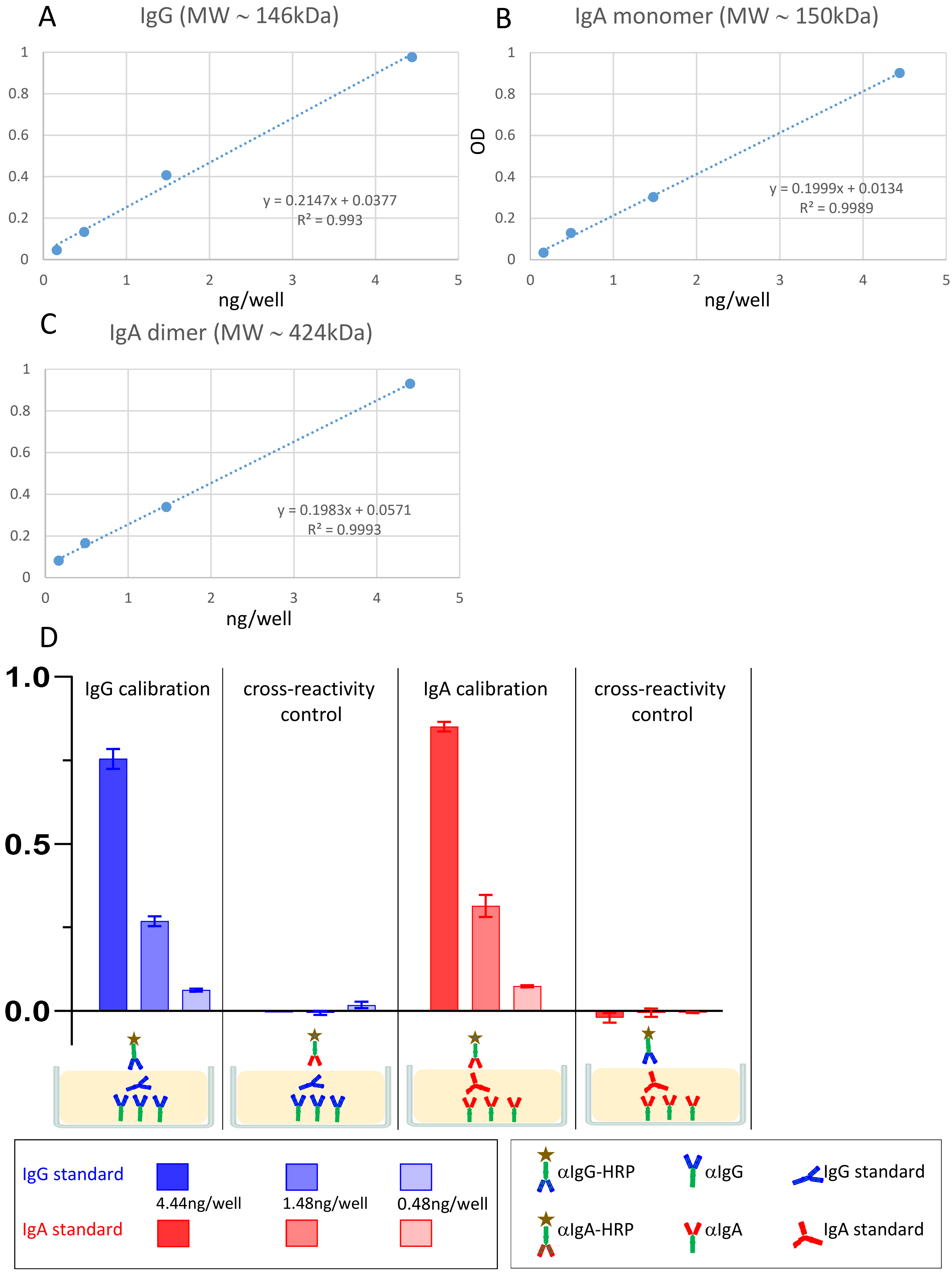

Figure S2

Isotype specific OD-to-mole transformation. (A-C) Sandwich capturing ELISA for selective quantification of total immunoglobulin isotypes (IgG and IgA). Monomeric IgG, IgA and dimeric IgA standards were applied to the plate to transform the OD values to their molar equivalents, shown are standard dilution curves used for extrapolation in capture ELISA format. (D) Shown are control experiments to assess the specificity of isotype capturing and isotype detection antibodies in ELISA assays, as depicted in diagrams above the bars.

Figure S3

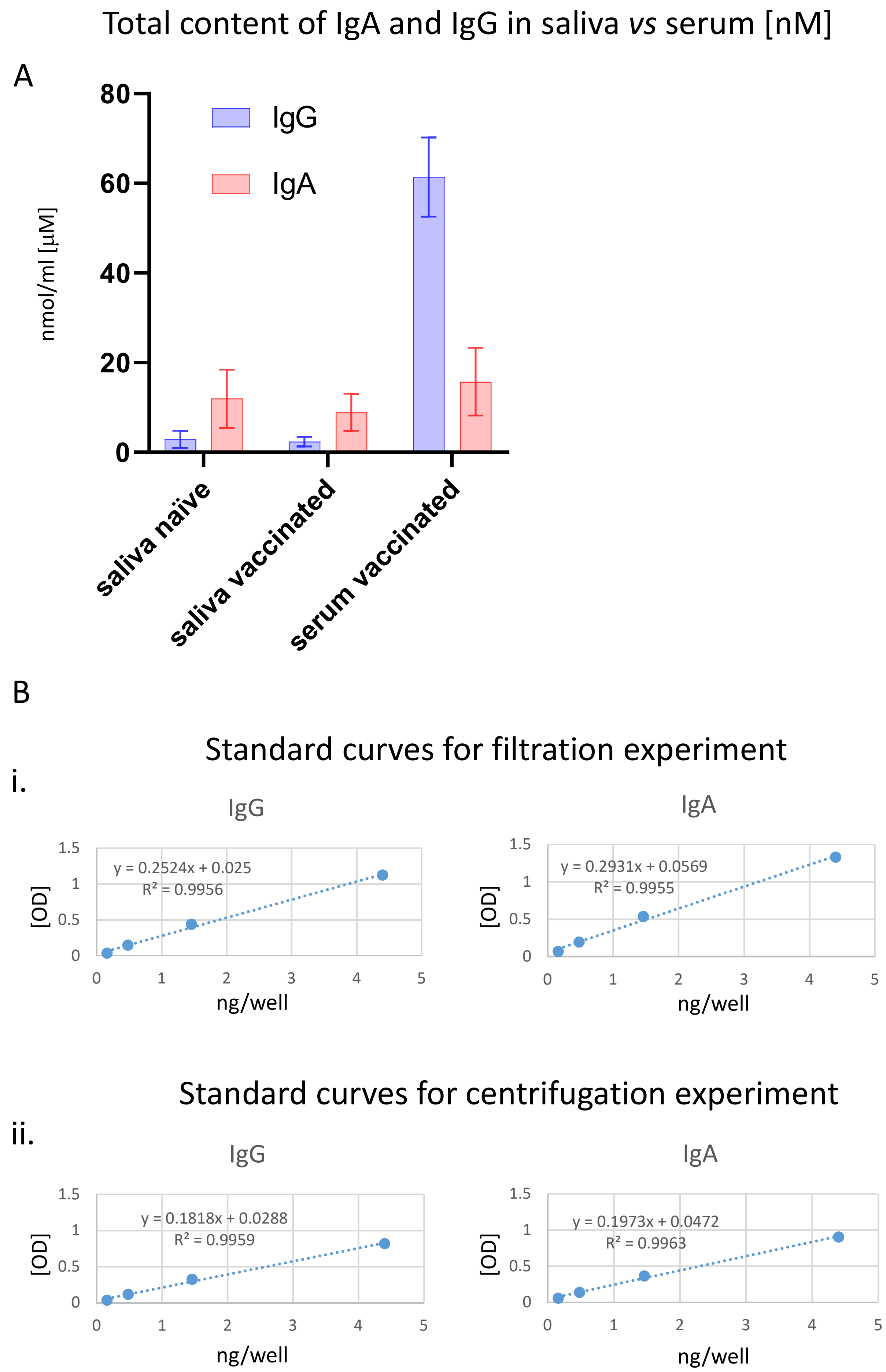
Total content of IgA and IgG in saliva *vs* serum [nM]
A
IgG
IgA
nmol/ml [µM]
80
60
40
20
0
saliva naïve
saliva vaccinated
serum vaccinated
B
Standard curves for filtration experiment
i.
IgG
y = 0.2524x + 0.025
R² = 0.9956
[OD]
ng/well
IgA
y = 0.2931x + 0.0569
R² = 0.9955
[OD]
ng/well
Standard curves for centrifugation experiment
ii.
IgG
y = 0.1818x + 0.0288
R² = 0.9959
[OD]
ng/well
IgA
y = 0.1973x + 0.0472
R² = 0.9963
[OD]
ng/well

Figures S3

Molar evaluation of the total immunoglobulin content confirms different isotype stoichiometry in saliva *versus* serum. (A) Molar IgA and IgG content were specifically determined in serum and saliva using sandwich ELISA as depicted in Figure 2B (see Methods for details). Tested samples included: naïve saliva (N=13), vaccinated saliva (N=22), vaccinated sera (N=13). IgG is the predominant isotype in serum, while IgA is predominant in saliva in accordance with previously published data. (B) Isotype specific OD-to-mole transformation for saliva samples presented in Figure 3 (see methods for details). Shown are standard dilution curves used for extrapolation in capture ELISA format. Extrapolated molar concentration used to evaluate recovery yields for soluble anti-RBD IgG and IgA from saliva samples upon (i) centrifugation and (ii) filtration are presented in Table S6.

Figure S4

A

Depletion of immunoglobulin isotypes IgA *vs* IgG in saliva

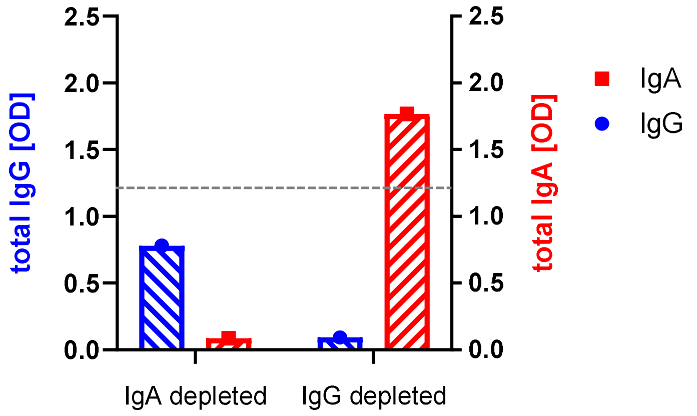

B

Specific detection of dimeric IgA with anti-SC antibody

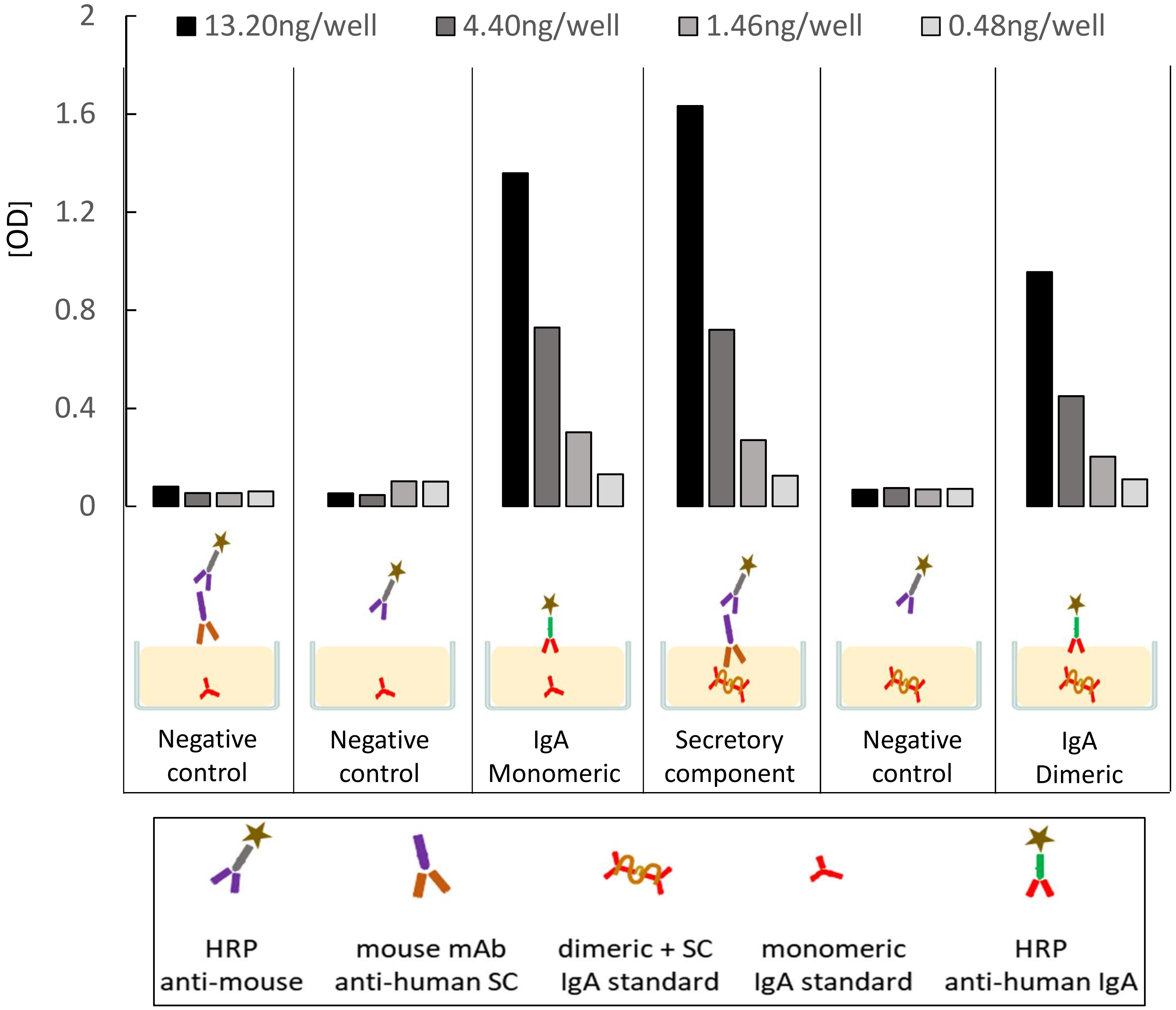

Figure S4

C Standard curves for pure human IgA dimer

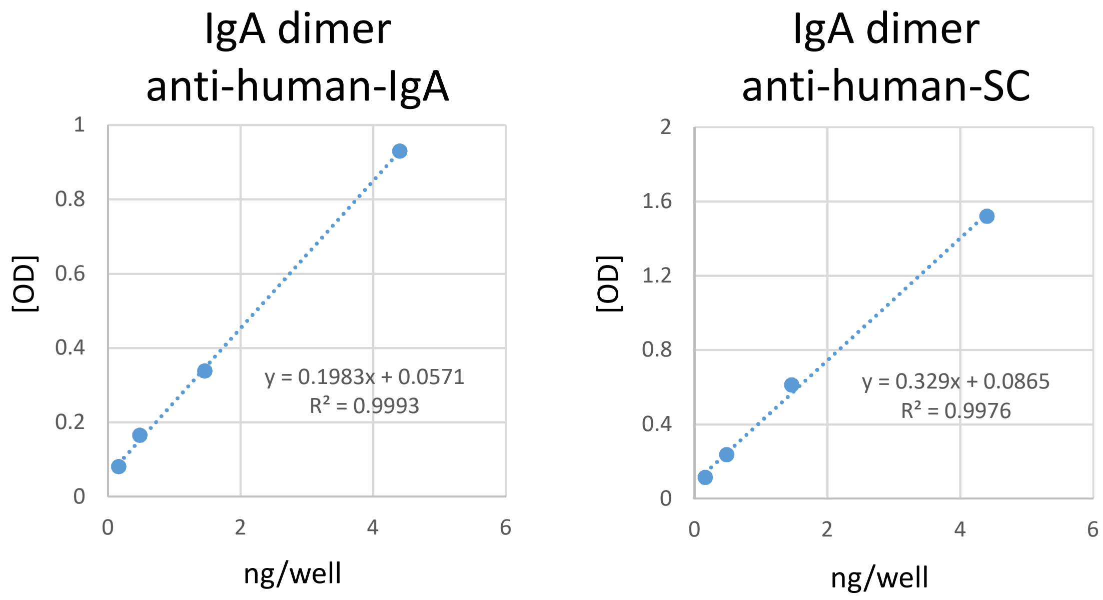

Figures S4

Studies of Abs in saliva of vaccinees. (A) Completeness of IgG and IgA selective depletions from pooled saliva samples used for neutralization experiments presented in Figure 4A. Left-side bars (IgA-depleted) samples were measured by sandwich ELISA for total IgG (blue column on the left) and for IgA (red column on the left). Right-side bars (IgG depleted) samples were measured by sandwich ELISA for total IgG (blue column on the right) and IgA (red column on the right), see methods for further details. IgG and IgA values are represented on separate ordinate axes (indicated), as explained in the main text. Horizontal dashed line indicates saturation level of ELISA measurement. Total immunoglobulins were measured in this experiment in saturated conditions to confirm the completeness of the depletion. (B) Specific detection of '*bona-fide*' dimeric IgA of mucosal origin bound to Secretory Component (SC) using anti-SC antibody (see methods and diagrams in Figure 4B, C for further details). The experimental details are depicted in diagrams below the corresponding bars. Serial dilutions of commercial standards: (i) monomeric human IgA purified from serum and (ii) dimeric secretory human IgA from colostrum were used as indicated. Graphical legend explains the pictogram identity. (C) Standard curves used for OD to molar transformations in the experiment presented in Figure 4D. Dimeric secretory human IgA from colostrum was used as a standard for detection with anti-human-IgA and anti-human-SC antibodies as indicated.

Figure S5 Expanded standard curves

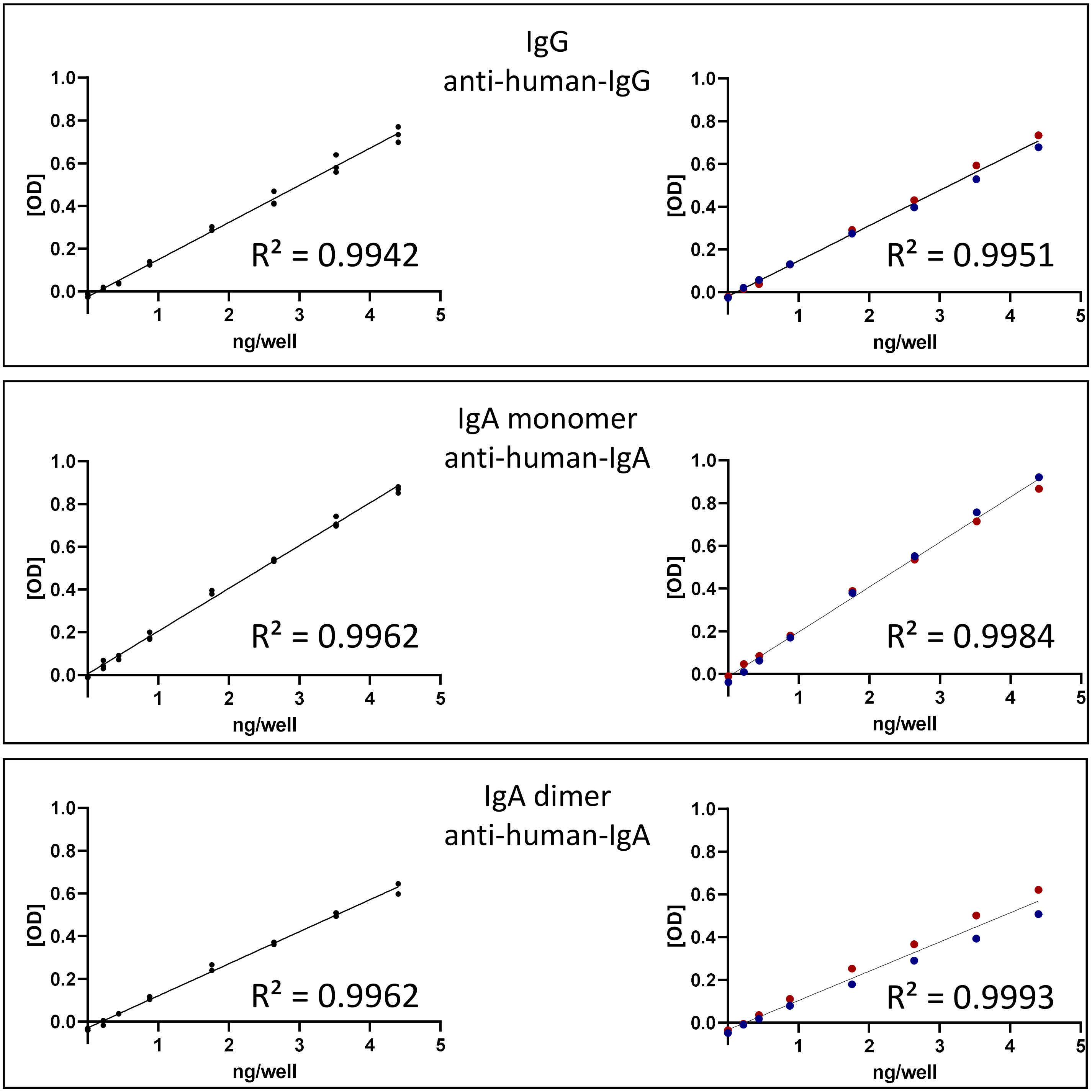

Figures S5

Expanded standard curves. Expanded standard dilution curves assess the robustness of the quantification method used for OD to molar transformation for comparative analysis of IgG and IgA isotypes in different biological fluids. Expanded curves include 8 consequent concentrations of the IgG, monomeric IgA and dimeric IgA standards, as indicated (see materials and methods for more details). Left panels present technical replicates performed using independent dilutions of commercial standards from the stock, performed on the same day (for IgG and monomeric IgA n=3, for dimeric IgA n=2). Right panels present two independent experiments performed on different days with independently prepared standard dilutions, blue and red. Each of the experiments in the right panels was performed in at least 2 or 3 technical replicates. The averaged technical replicates are shown for each independent experiment. R squared simple linear regression was calculated in GraphPad Prism for each curve using averages of the presented experimental repeats for the corresponding IgG and IgA standard dilutions.

Figure S6

Model: Molar neutralizing potential in circulation and mucosa

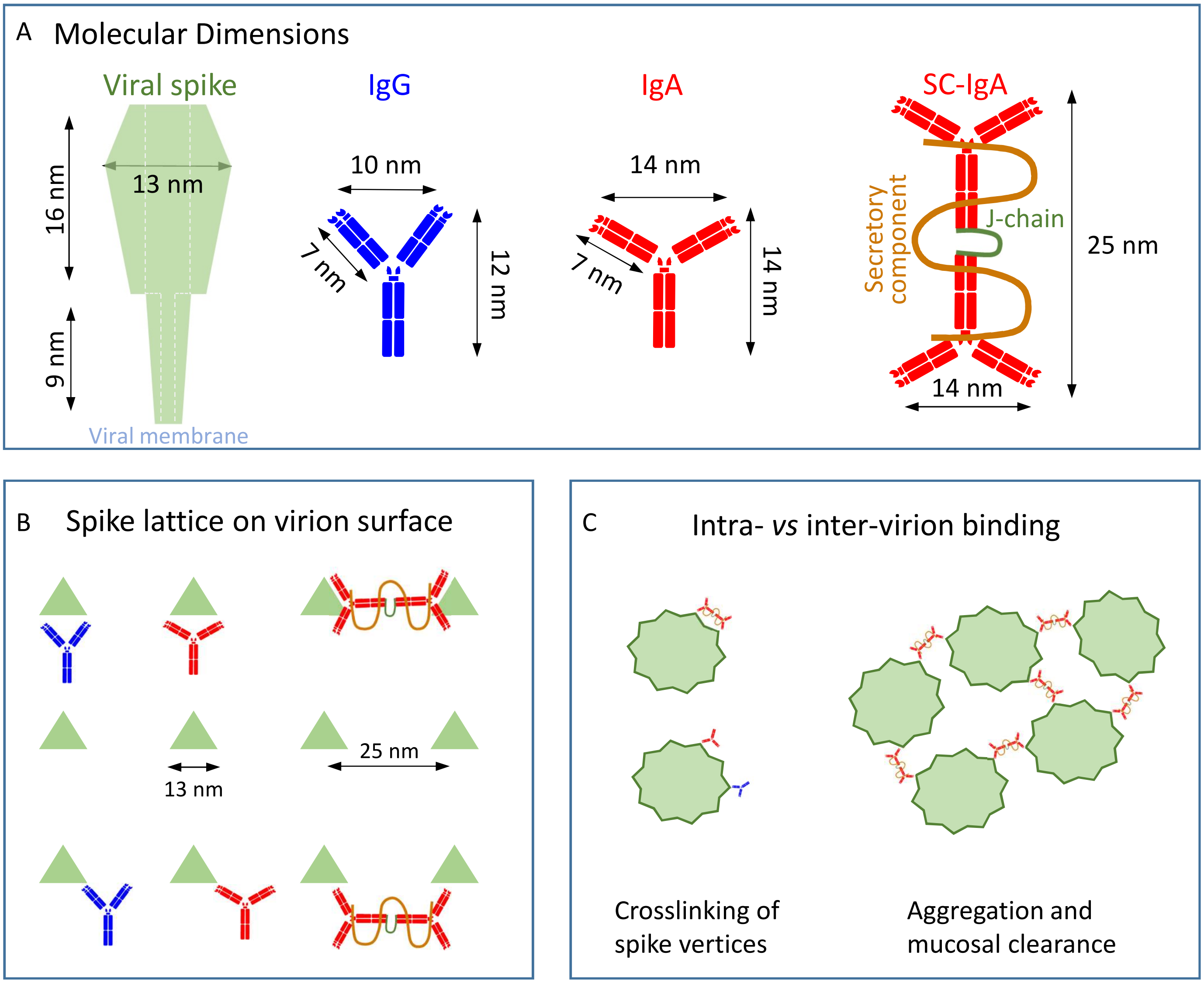

Figure S6

Model: Molar neutralizing potential in circulation and mucosa - '*GedankenExperiment*' to explain protective outcome. (A) Known dimensions of SARS-CoV-2 spike protein trimer next to dimensions of circulatory and mucosal immunoglobulins. The extension and flexibilities of IgA arms are illustrated by wider angularity of the Fabs for the monomer. The longitudinal extension of the dimeric SC-IgA is represented in the right panel. (C) Lattice density of trimeric spike vertices, represented by green triangular surface projections of a viral antigen and their coverage by immunoglobulins are shown (see text for details). (D) Illustration of protective outcomes: SC-IgA versus IgG surface interaction areas. (E) Illustration of the interaction modalities in the context of the virus particle: intra-virion binding of SC-IgA and monomeric IgG and IgA (left panel) and inter-virion aggregation by SC-IgA (right panel). SC-IgA in mucosal surfaces and secretions exist in different polymeric forms. The most studied are tetravalent dimers (schematically depicted above) and octavalent tetramers, although higher oligomers were also reported. The specific degree of oligomerization would have a significant impact on the molecular dimensions and 3D-architecture of SC-IgA and on its avidity properties, both of which would affect functionality in terms of neutralization and of aggregation mediated mucosal clearance.

Table S1

Category	Participant	Days pv1	Days pv2	Days between disease onset and blood collection	Age group	Gender	Anti-RBD IgG (OD)	Anti-RBD IgA (OD)
preCOVID	1000	na	na	na			0.078	0.076
preCOVID	1001	na	na	na			0.085	0.077
preCOVID	1002	na	na	na			0.072	0.067
preCOVID	1003	na	na	na			0.103	0.073
preCOVID	1004	na	na	na			0.090	0.067
preCOVID	1005	na	na	na			0.073	0.067
preCOVID	1006	na	na	na			0.084	0.067
preCOVID	1007	na	na	na			0.084	0.068
preCOVID	1008	na	na	na			0.088	0.064
preCOVID	1009	na	na	na			0.074	0.061
preCOVID	1010	na	na	na			0.112	0.070
preCOVID	1011	na	na	na			0.093	0.073
preCOVID	1012	na	na	na			0.081	0.068
preCOVID	1013	na	na	na			0.088	0.068
preCOVID	1014	na	na	na			0.074	0.082
preCOVID	1015	na	na	na			0.103	0.069
preCOVID	1016	na	na	na			0.078	0.062
preCOVID	1017	na	na	na			0.081	0.074
preCOVID	1018	na	na	na			0.076	0.062
preCOVID	1019	na	na	na			0.090	0.065
preCOVID	1020	na	na	na			0.074	0.062
preCOVID	1021	na	na	na			0.073	0.069
preCOVID	1022	na	na	na			0.083	0.068
preCOVID	1023	na	na	na			0.079	0.067
preCOVID	1024	na	na	na			0.092	0.065
preCOVID	1025	na	na	na			0.082	0.062
preCOVID	1026	na	na	na			0.112	0.065
preCOVID	1027	na	na	na			0.119	0.066
preCOVID	1028	na	na	na			0.077	0.069
preCOVID	1029	na	na	na			0.106	0.068
preCOVID	1030	na	na	na			0.076	0.070
preCOVID	1031	na	na	na			0.105	0.067
preCOVID	1032	na	na	na			0.078	0.065
preCOVID	1033	na	na	na			0.077	0.065
preCOVID	1034	na	na	na			0.071	0.062
preCOVID	1035	na	na	na			0.089	0.066
preCOVID	1036	na	na	na			0.074	0.065
preCOVID	1037	na	na	na			0.084	0.072
preCOVID	1038	na	na	na			0.077	0.066
preCOVID	1039	na	na	na			0.077	0.063
preCOVID	1040	na	na	na			0.099	0.068
preCOVID	1041	na	na	na			0.100	0.073
preCOVID	1042	na	na	na			0.077	0.057
preCOVID	1043	na	na	na			0.090	0.061
preCOVID	1044	na	na	na			0.074	0.067
preCOVID	1045	na	na	na			0.078	0.073
preCOVID	1046	na	na	na			0.087	0.078
preCOVID	1047	na	na	na			0.075	0.062
preCOVID	1048	na	na	na			0.115	0.094
preCOVID	1049	na	na	na			0.090	0.079
preCOVID	1050	na	na	na			0.095	0.067

Table S1

Category	Participant	Days pv1	Days pv2	Days between disease onset and blood collection	Age group	Gender	Anti-RBD IgG (OD)	Anti-RBD IgA (OD)
vac	1	32	11	na	40-50	m	13.192	0.609
vac	2	45	24	na	40-50	f	29.487	0.560
vac	3	30	9	na	70-75	m	25.152	2.564
vac	4	51	30	na	60-65	m	38.551	1.719
vac	5	31	10	na	50-60	f	37.897	2.946
vac	6	31	10	na	50-60	f	18.922	2.943
vac	7	40	19	na	20-40	f	35.532	0.658
vac	8	84	63	na	40-50	m	9.279	0.296
vac	10	35	14	na	60-65	f	43.791	0.894
vac	11	35	14	na	65-70	m	22.785	0.744
vac	12	35	14	na	20-40	f	46.216	1.457
vac	13	36	13	na	50-60	f	11.637	0.317
vac	14*	42	21	na	20-40	f	7.524	0.154
vac	15*	63	42	na	60-65	m	10.010	0.228
vac	16	62	41	na	65-70	m	13.882	0.315
vac	17	63	42	na	60-65	f	9.707	0.121
vac	18	42	21	na	65-70	f	24.227	0.251
convalescent	500	na	na				27.999	3.075
convalescent	501	na	na				2.177	0.937
convalescent	502	na	na				16.197	0.801
convalescent	503	na	na				1.075	0.198
convalescent	504	na	na				1.737	0.436
convalescent	505	na	na				20.085	5.497
convalescent	506	na	na				0.285	0.083
convalescent	507	na	na				2.160	0.170
convalescent	508	na	na				1.853	0.259
convalescent	509	na	na				5.148	0.308
convalescent	510	na	na				2.721	0.324
convalescent	511	na	na				2.535	0.090
convalescent	512	na	na				1.363	0.234
convalescent	513	na	na				2.464	0.172
convalescent	514	na	na				1.199	0.185
convalescent	515	na	na				1.225	0.407
convalescent	516	na	na				1.404	0.277
convalescent	517	na	na				2.999	0.297
convalescent	19	na	na	36	20-40	m	0.795	0.119
convalescent	21	na	na	27	20-40	m	0.342	0.162
convalescent	22	na	na	14	50-60	f	1.904	0.780
convalescent	23	na	na	89	20-40	m	0.266	0.075

Table S1

Cohort details related to figure 1A. Serum samples from pre-COVID, vaccinated, and convalescent participants were assayed for anti-RBD IgG and anti-RBD IgA and presented as group in main figure. Here, individual values for each participant are presented. Total number of serum samples (N=90) were taken from 90 participants (P=90) were assayed in the presented sub-cohort. (*) indicates individuals with special immune background, routinely receiving immune suppression treatment (anti-TNF-alpha). Days pv1 and days pv2 indicate time interval between the respective vaccine dose and the serum sampling. “rec” corresponds to recovered, convalescent, participants.

Table S2

Participant	Serial sample	Days pv1	Days pv2	Age	Gender
1	I	0	na	40-50	m
1	II	7	na	40-50	m
1	III	17	na	40-50	m
1	IV	24	3	40-50	m
1	V	32	11	40-50	m
1	VI	41	20	40-50	m
1	VII	48	27	40-50	m
1	VIII	60	39	40-50	m
1	IX	84	63	40-50	m
1	X	108	87	40-50	m
1	XI	179	158	40-50	m
2	I	0	na	40-50	f
2	II	3	na	40-50	f
2	III	7	na	40-50	f
2	IV	14	na	40-50	f
2	V	21	na	40-50	f
2	VI	24	3	40-50	f
2	VII	28	7	40-50	f
2	VIII	45	24	40-50	f
2	IX	56	35	40-50	f
2	X	84	63	40-50	f
2	XI	108	87	70-75	f
2	XII	179	158	40-50	f
3	I	0	na	70-75	m
3	II	14	na	70-75	m
3	III	30	9	70-75	m
3	IV	48	27	70-75	m
3	V	116	95	70-75	m
3	VI	186	165	70-75	m
4	I	0	na	60-65	m
4	II	7	na	60-65	m
4	III	17	na	60-65	m
4	IV	24	3	60-65	m
4	V	41	20	60-65	m
4	VI	51	30	60-65	m
4	VII	56	35	60-65	m
4	VIII	84	63	60-65	m
4	IX	108	87	60-65	m
4	X	179	158	60-65	m
5	I	7	na	50-60	f
5	II	17	na	50-60	f
5	III	24	3	50-60	f
5	IV	31	10	50-60	f
5	V	41	20	50-60	f
5	VI	63	42	50-60	m
5	VII	84	63	50-60	f
5	VIII	108	87	50-60	f
5	IX	179	158	50-60	f
6	I	7	na	50-60	f
6	II	17	na	50-60	f
6	III	31	10	50-60	f
6	IV	41	20	50-60	f
6	V	60	39	50-60	f
6	VI	111	90	50-60	f
7	I	9	na	20-40	f
7	II	20	na	20-40	f
7	III	24	3	20-40	f
7	IV	40	19	20-40	f
7	V	68	47	20-40	f
7	VI	103	82	20-40	f
7	VII	189	168	20-40	f
8	I	24	3	40-50	m
8	II	84	63	40-50	m
8	III	108	87	40-50	m
8	IV	189	168	40-50	m
9	I	26	5	70-75	m
10	I	35	14	60-65	f
11	I	35	14	65-70	m
12	I	35	14	20-40	f
13	I	36	13	50-60	f
13	II	56	33	50-60	f
13	III	197	174	50-60	f
14*	I	42	21	20-40	f
15	I	42	21	65-70	f
16	I	62	41	65-70	m
17	I	63	42	60-65	f
18*	I	63	42	60-65	m

Table S2

Longitudinal sampling of anti-RBD IgG and IgA in serum - cohort details, related to figure S1B.

Total number of serum samples (N=76 for IgG and N=75 for IgA) in the presented sub-cohort were collected kinetically from 18 participants (P=18). Subjects with serial sampling are indicated in ‘serial sample column’, by consequent numbering. (*) indicates individuals with special immune background, routinely receiving immune suppression treatment (anti-TNF-alpha). Days pv1 and days pv2 indicate time interval between the respective vaccine dose and the serum sampling. (#) indicates the sample for which only anti-RBD IgG was assayed. See Figures 1B and S2D for the serological anti-RBD IgG and anti-RBD IgA data and the corresponding graphical representations.

Table S3:

Category	Participant	Serial sample	Days pv1	Days pv2	Age group	Gender	Serial sampling in Table S1 for Fig. 1A
naïve	13	a	na	na	50-60	f	
vac	13	c	36	13	50-60	f	13 I
vac	13	d	56	33	50-60	f	13 II
vac	14*	a	42	21	20-40	f	14 I
vac	15*	a	63	42	60-65	m	15 I
naïve	16	a	na	na	65-70	m	
vac	16	b	62	41	65-70	m	16 I
vac	17	a	38	17	40-50	m	
vac	18	a	42	21	65-70	f	18 I
rec	19	a	na	na	20-40	m	23
rec	19	b	na	na	20-40	m	36
rec	19	c	na	na	20-40	m	53
rec	19	d	na	na	20-40	m	212
rec	19	e	na	na	20-40	m	308
rec	20	a	na	na	50-60	f	101
naïve	21	a	na	na	20-40	m	
rec	21	b	na	na	20-40	m	27
rec vac	21	c	0	na	20-40	m	64
rec vac	21	d	12	na	20-40	m	76
rec vac	21	e	21	0	20-40	m	85
rec vac	21	f	24	3	20-40	m	88
rec vac	21	g	32	11	20-40	m	96
rec vac	21	h	84	63	20-40	m	148
rec	22	a	na	na	50-60	f	14
rec	22	b	na	na	50-60	f	84
rec vac	22	c	2	na	50-60	f	101
rec vac	22	d	13	na	50-60	f	112
rec vac	22	e	22	0	50-60	f	121
rec	23	a	na	na	20-40	m	33
rec	23	b	na	na	20-40	m	89
rec vac	23	c	14	na	20-40	m	106
rec vac	23	a	21	0	20-40	m	113

Table S3

Pearson correlation cohort details related to figure S1C: including indication of longitudinal sampling for Pearson correlation between our quantitative test and the ARCHITECT (Abbott, Illinois, U.S.A.) for anti-RBD IgG. Serum samples from naïve, vaccinated, recovered and recovered-vaccinated (vaccinated post-recovery) participants are presented. Total number of serum samples in the presented sub-cohort (N=97) were collected kinetically from 23 participants (P=23). Subjects with serial sampling are indicated in 'serial sample column'. (*) indicates an individual with special immune background, routinely receiving immune suppression treatment (anti-TNF-alpha). Days pv1 and days pv2 indicate time interval between the respective vaccine dose and the serum sampling. "rec" corresponds to recovered participants and "rec vac" to participants that were recovered from disease and further vaccinated. Sample numbering is coded and presented according to combined cohort table.

Table S4

Days pv1	Days pv2	Anti-RBD IgG (nM)	Anti-RBD IgA (nM)	Total IgG (μM)	Total IgA (μM)	% anti-RBD IgG of total IgG	% anti-RBD IgA of total IgA	Age	Gender
17	na	11.9	0.0	75.3	26.8	0.02	0.00	40-50	m
17	na	302.5	63.2	48.6	10.5	0.62	0.60	50-60	f
17	na	49.8	156.3	63.7	16.9	0.08	0.92	50-60	f
20	na	174.6	18.5	67.5	12.7	0.26	0.15	20-40	f
24	3	141.8	34.7	73.7	14.9	0.19	0.23	40-50	f
24	3	215.9	56.8	70.0	6.1	0.31	0.93	40-50	m
24	3	127.8	21.0	52.2	28.3	0.24	0.07	60-65	m
27	6	156.7	23.9	61.1	4.4	0.26	0.54	70-75	m
30	6	765.1	ND*	66.2	21.9	1.16	ND*	70-75	m
34	13	297.5	18.7	55.2	7.5	0.54	0.25	50-60	f
35	14	1419.2	71.1	56.8	18.0	2.50	0.40	60-65	f
35	14	436.5	48.2	50.0	15.0	0.87	0.32	65-70	m
38	17	151.9	9.5	58.1	21.4	0.26	0.04	40-50	m

Table S4

Anti-RBD IgG and IgA *vs* total IgG and IgA in serum of BNT162b2 vaccinees expressed in molar concentration (nM and μM, as indicated), see figure 2C and 2D for graphical representation and statistical analysis.

Table S5 - Part I

Category	Participant	Serial sample	Days pv1	Days pv2	Anti-RBD IgA (nM)	Age	Gender
naïve	1	I	na	na	0.214	20-40	m
naïve	1	II	na	na	0.597	20-40	m
naïve	1	III	na	na	0.322	20-40	m
naïve	2	I	na	na	0.104	20-40	m
naïve	2	II	na	na	0.119	20-40	m
naïve	2	III	na	na	0.303	20-40	m
naïve	3	I	na	na	0.058	20-40	f
naïve	3	II	na	na	0.115	20-40	f
naïve	3	III	na	na	0.149	20-40	f
naïve	4	I	na	na	0.554	20-40	m
naïve	5	I	na	na	0.435	20-40	m
naïve	6	I	na	na	0.375	20-40	f
naïve	7	I	na	na	0.008	20-40	f
naïve	7	II	na	na	0.328	20-40	f
naïve	7	III	na	na	0.011	20-40	f
naïve	8	I	na	na	0.360	20-40	m
naïve	8	II	na	na	0.372	20-40	m
naïve	8	III	na	na	0.240	20-40	m
naïve	9	I	na	na	0.133	20-40	f
naïve	10	I	na	na	0.386	40-50	m
naïve	11	I	na	na	0.319	20-40	m
vac	12	I	41	20	0.467	40-50	m
vac	12	II	60	39	0.454	40-50	m
vac	12	III	84	63	0.358	40-50	m
vac	12	IV	108	87	0.511	40-50	m
vac	12	V	142	121	0.118	40-50	m
vac	12	VI	146	125	0.529	40-50	m
vac	12	VII	157	136	0.038	40-50	m
vac	12	VIII	171	150	0.018	40-50	m
vac	12	IX	190	169	0.043	40-50	m
vac	12	XI	195	174	0.036	40-50	m
vac	12	XII	144	123	0.580	40-50	m
vac	12	XIII	169	148	0.037	40-50	m
vac	12	XIIII	179	158	0.051	40-50	m
vac	13	I	49	28	0.214	40-50	f
vac	13	II	56	35	0.085	40-50	f
vac	13	III	84	63	0.447	40-50	f
vac	13	IV	108	87	0.208	40-50	f
vac	13	V	141	120	0.047	40-50	f
vac	13	VI	143	122	0.263	40-50	f
vac	13	VII	143	122	0.176	40-50	f
vac	13	VIII	143	122	0.073	40-50	f
vac	13	IX	147	126	0.226	40-50	f
vac	13	X	154	133	0.345	40-50	f
vac	13	XI	171	150	0.231	40-50	f
vac	14	I	41	20	0.716	60-65	m
vac	14	II	56	35	0.718	60-65	m
vac	14	III	84	63	0.813	60-65	m
vac	14	IV	108	87	0.601	60-65	m
vac	14	V	179	158	0.320	60-65	m
vac	14	VI	171	150	0.163	60-65	m
vac	15	I	63	42	0.603	50-60	f
vac	15	II	63	42	0.583	50-60	f
vac	15	III	84	63	0.701	50-60	f
vac	15	IV	108	87	1.103	50-60	f
vac	15	V	179	158	0.060	50-60	f

Table S5 - Part I

Category	Participant	Serial sample	Days pv1	Days pv2	Anti-RBD IgA (nM)	Age	Gender
vac	16	I	41	20	0.223	50-60	f
vac	16	II	60	39	0.203	50-60	f
vac	16	III	111	90	0.673	50-60	f
vac	17	I	186	165	0.041	70-75	m
vac	18	I	62	41	0.490	65-70	m
vac	19*	I	42	21	0.257	20-40	f
vac	20	I	103	82	0.218	20-40	f
vac	20	II	103	82	0.212	20-40	f
vac	20	III	139	118	0.471	20-40	f
vac	22	II	84	63	0.642	40-50	m
vac	22	III	108	87	0.747	40-50	m
vac	23	I	175	154	0.108	20-40	f
vac	24	I	204	181	0.187	50-60	f
vac	25	I	176	155	0.005	20-40	f
vac	26	I	178	157	0.326	20-40	m
vac	27	I	176	155	0.080	20-40	f
vac	28	I	119	98	0.787	40-50	m

Table S5 - Part II

Category	Participant	Serial sample	Days pv1	Days pv2	Anti-RBD IgA (nM)	Age	Gender	Days between disease onset and blood collection	Days between disease onset and vaccination
rec	29	I	na	na	0.720	20-40	f	135	
rec	30	I	na	na	0.606	20-40	m	380	
rec vac	31	I	61	39	1.1#	50-60	f	160	99
rec vac	32	I	52	31	0.816	20-40	m	116	64
rec vac	32	II	84	63	0.421	20-40	m	148	64
rec vac	32	III	109	88	0.373	20-40	m	173	64
rec vac	32	IV	123	102	0.417	20-40	m	187	30
rec vac	32	V	157	136	0.106	20-40	m	221	64
rec vac	33	I	82	61	0.189	20-40	m	174	92

Table S5
Saliva cohort details including indication of longitudinal sampling, see figure 3A for saliva anti-RBD IgA data and the corresponding graphical representations of naïve and vaccinated participants. Total number of saliva samples in the presented sub-cohort (N=82) were collected longitudinally from 33 participants (P=33).
Part I: Samples from naïve and vaccinated individuals. Samples (N) and participants (P) categorised: unvaccinated (naïve), N(21)/P(11); 40-65d pv1, N(13)/P(8); 80-115d pv1 N(14)/P(8); 135-160d pv1 N(12)/P(3); 170-205d pv1 N(15)/P(10), see Fig.3A in the main text. Subjects with serial sampling are indicated in 'serial sample column', by consequent numbering. (*) indicates individual with special immune background, routinely receiving immune suppression treatment (anti-TNF-alpha). (#) indicates value above the linear detection range. Days pv1 and days pv2 indicate time interval between the respective vaccine dose and saliva sampling.
Part II: Samples of participants included in the survey that stayed unvaccinated after recovery or recovered and further vaccinated: "rec" corresponds to recovered participants and "rec vac" to participants that were recovered from disease and further vaccinated.

Table S6

i.

Days pv1	Days pv2	Anti-RBD IgG (nM) before centrifugation	Anti-RBD IgG (nM) post-centrifugation	Anti-RBD IgA (nM) before centrifugation	Anti-RBD IgA (nM) post-centrifugation
naive	naive	0.00	0.00	0.43	0.07
56	35	1.41	1.68	1.56	1.38
108	87	0.23	0.18	0.86	1.05
108	87	0.08	0.10	0.41	0.16
108	87	0.39	0.31	1.56	1.50
123	102	0.14	0.13	1.28	1.26
142	121	0.10	0.08	0.19	0.04

ii.

Days Pv1	Days pv2	Anti-RBD IgG (nM) before filtration	Anti-RBD IgG (nM) post filtration	Anti-RBD IgA (nM) before filtration	Anti-RBD IgA (nM) post filtration
naive	naive	0.00	0.00	0.17	0.24
56	35	1.99	1.99	1.36	1.50
108	87	0.03	0.04	0.21	0.25
108	87	0.46	0.52	1.67	1.48
123	102	0.10	0.12	0.41	0.50

Table S6
Recovery yields of soluble anti-RBD IgG and IgA from saliva samples tested upon Part I: centrifugation and **Part II:** filtration. The samples used in panel (ii) were first centrifuged and then either directly assayed or further centrifuged, as indicated.

Table S7

Integrated serum cohort table: Information on serum samples and serum cohort demographics from pre-COVID, vaccinated, and convalescent participants used in the study is integrated in the table. Additional information on the cohort is described in material and methods. The table is presented in **Table S7.xls** format for the purpose of convenient cross-reference search of samples used along the Figures/Tables presented in the manuscript. (*) indicates individuals with special immune background, routinely receiving immune suppression treatment (anti-TNF-alpha). Days pv1 and days pv2 indicate time interval between the respective vaccine dose and the serum sampling. (&) indicates individuals suspected, but not confirmed undiagnosed SARS-CoV-2 by anti-N, IgG seropositivity. Individuals with the collected saliva samples are indicated by the corresponding sample index of the saliva cohort. Longitudinal sampling of the participants is indexed alphabetically. “rec” corresponds to recovered, convalescent, participants. In this category of previously documented COVID-19 patients we included only cases confirmed via PCR test according to clinical definition of National Ministry of Health, Israel. One specific patient recruited in the cohort (marked as recovered and highlighted by # in Tables S7 and S8), was not tested by PCR during the symptomatic phase of the COVID-19 disease, but was subsequently confirmed as serologically positive by 3 independent tests (our anti-RBD and anti-N ELISA assays, as well as clinical anti-RBD IgG test, ARCHITECT (Abbott, Illinois, U.S.A.)). This particular patient was not vaccinated.

Table S8

Information on saliva samples and saliva cohort demographics from pre-COVID, vaccinated, and convalescent participants used in the study is integrated in the table. Additional information on the cohort is described in material and methods. The table is presented in **TableS8.xls** format for the purpose of convenient cross-reference search of samples used along the Figures/Tables presented in the manuscript. (*) indicates individuals with special immune background, routinely receiving immune suppression treatment (anti-TNF-alpha). Days pv1 and days pv2 indicate time interval between the respective vaccine dose and the serum sampling. (&) indicates individuals suspected, but not confirmed undiagnosed SARS-CoV-2 by anti-N, IgG seropositivity. In the category of previously documented COVID-19 patients we included only cases confirmed via PCR test according to clinical definition of National Ministry of Health, Israel. One specific patient recruited in the cohort (marked as recovered and highlighted by # in Tables S7 and S8), was not tested by PCR during the symptomatic phase of the COVID-19 disease, but was subsequently confirmed as serologically positive by 3 independent tests (our anti-RBD and anti-N ELISA assays, as well as clinical anti-RBD IgG test, ARCHITECT (Abbott, Illinois, U.S.A.)). This particular patient was not vaccinated.
